# Bayesian multi-task learning for decoding multi-subject neuroimaging data

**DOI:** 10.1016/j.neuroimage.2014.02.008

**Published:** 2014-05-15

**Authors:** Andre F. Marquand, Michael Brammer, Steven C.R. Williams, Orla M. Doyle

**Affiliations:** Department of Neuroimaging, Institute of Psychiatry, De Crespigny Park, London SE5 8AF, United Kingdom

**Keywords:** Multi-task learning, Multi-output learning, Transfer learning, Gaussian process, Functional magnetic resonance imaging, Repeated measures, Pattern recognition, Machine learning, Decoding, Mixed effects

## Abstract

Decoding models based on pattern recognition (PR) are becoming increasingly important tools for neuroimaging data analysis. In contrast to alternative (mass-univariate) encoding approaches that use hierarchical models to capture inter-subject variability, inter-subject differences are not typically handled efficiently in PR. In this work, we propose to overcome this problem by recasting the decoding problem in a multi-task learning (MTL) framework. In MTL, a single PR model is used to learn different but related “tasks” simultaneously. The primary advantage of MTL is that it makes more efficient use of the data available and leads to more accurate models by making use of the relationships between tasks. In this work, we construct MTL models where each subject is modelled by a separate task. We use a flexible covariance structure to model the relationships between tasks and induce coupling between them using Gaussian process priors. We present an MTL method for classification problems and demonstrate a novel mapping method suitable for PR models. We apply these MTL approaches to classifying many different contrasts in a publicly available fMRI dataset and show that the proposed MTL methods produce higher decoding accuracy and more consistent discriminative activity patterns than currently used techniques. Our results demonstrate that MTL provides a promising method for multi-subject decoding studies by focusing on the commonalities between a group of subjects rather than the idiosyncratic properties of different subjects.

## Highlights

• In mass-univariate analysis, mixed effects models can capture subject variability.
• In pattern recognition (PR), subject variability is usually not modelled explicitly.
• Multi-task learning (MTL) is proposed to accommodate subject variability in PR.
• The proposed approach improves predictive accuracy and pattern reproducibility.
• A novel brain mapping approach is also proposed for MTL and existing PR models.

## Introduction

Pattern recognition (PR) methods are becoming increasingly important tools for neuroimaging data analysis and are complementary to more conventional mass-univariate analysis methods based on the general linear model (GLM; Friston et al. (1995)). Mass-univariate methods, or encoding models (Naselaris et al., 2011), are well suited to mapping focal, group level associations between experimental variables and brain structure or function. On the other hand, PR methods, or decoding models, aim to make predictions based on the spatial or spatiotemporal pattern within the data. In particular, PR methods have been useful for making predictions at the single subject level in clinical research studies (Orru et al., 2012) and for detecting neural activity patterns characteristic of instantaneous cognitive states (Norman et al., 2006).

Neuroimaging data are well-known to be characterised by substantial inter-subject variability, due to a range of factors including residual registration error, variations in inter-subject functional anatomy (Frost and Goebel, 2012; Morosan et al., 2001) and individual variations in the haemodynamic response (Aguirre et al., 1998; Handwerker et al., 2004). In a mass-univariate context, this variability has been historically tackled using hierarchical classical or Bayesian random- or mixed-effects models (e.g. Holmes and Friston (1998); Friston et al. (2005); Woolrich et al. (2004)), but in a PR context these individual differences are not usually dealt with efficiently. The two most common approaches for PR in multi-subject neuroimaging studies are: (i) training an individual classification or regression model for each subject (e.g. references in Norman et al. (2006)) or (ii) pooling data across subjects (e.g. Mourao-Miranda et al. (2005); Marquand et al. (2011); Brodersen et al. (2012); Grosenick et al. (2013)). Both these approaches are suboptimal; the first approach does not take advantage of similarities between different subjects and results in training PR models with a greatly reduced number of samples. The second method incorrectly assumes that all data are drawn from the same distribution and may lead to impaired predictive performance. A further problem is that most current PR approaches employed in neuroimaging provide no means of accommodating repeated measurements from the same subjects and again make the incorrect assumption that all data are independent and identically distributed.

In this work, we propose an alternative approach to accommodate the within- and between-subject covariance structure in neuroimaging data by recasting the decoding problem in a multi-task learning framework (MTL; Caruana (1997)). Multi-task learning is an emerging field of machine learning that aims to solve a number of related problems (“tasks”) simultaneously, taking into account the relationships between them. One of the key aims is to avoid learning each task from scratch and instead, MTL aims to extract more information from the data by sharing information between tasks. It is particularly beneficial in situations where only a small number of samples are available for each task, but other related tasks are available which share some salient properties (Bakker and Heskes, 2003; Evgeniou et al., 2005; Sheldon, 2008). In many cases MTL can lead to substantial improvements in predictive performance (Pan and Yang, 2010). In this work, we model the functional neuroimaging data for each subject as a separate task, induce coupling between them, and then estimate the optimal correlation structure for the tasks. In this way, we aim to learn a consistent pattern of activity across subjects, which is usually what is of primary interest in multi-subject neuroimaging studies. At the same time, this framework still allows flexibility to model the idiosyncratic properties of individual subjects and the ability to accommodate the statistical dependencies between scans (e.g. due to repeated measurements), since all the scans for individual subjects are grouped into tasks.

We propose to model the relationships between the tasks using a free-form covariance matrix and induce coupling between the tasks using Gaussian process (GP) priors (Bonilla et al., 2008). Under this framework, the task covariance matrix can be either specified in advance or estimated automatically from the data. This data-driven property is a crucial requirement for neuroimaging data, because it can be difficult to know in advance the extent of inter-subject variation functional anatomy in an experimental population. Gaussian process models are a flexible class of models for non-parametric regression and classification (Rasmussen and Williams, 2006). They are well-suited to neuroimaging data and hold advantages over alternative methods, including accurate quantification of uncertainty and elegant methods for automatic parameter optimisation. We have demonstrated in previous work that they are useful for whole-brain binary and multi-class classification of neuriomaging data, in addition to metric and ordinal regression (Doyle et al., 2013; Filippone et al., 2012; Marquand et al., 2010, 2013b).

In contrast to many other application domains where predictive performance is of primary interest, an important second objective of PR methods for neuroimaging is to quantify the contribution of different brain regions to the discriminative patterns that underlie the prediction. This is typically done by mapping model coefficients in the voxel space, which is also a useful method to assess the reproducibility of the spatial patterns under perturbations to the data (Strother et al., 2004). This formalises the intuition that we should prefer models that yield stable parameter values for different training datasets (i.e. are trustworthy). In view of these desiderata, we focus on linear models, which have an exact representation of model coefficients in the voxel space, although the MTL approach can be also be applied to non-linear models. We also present a novel brain mapping method for MTL and other PR models based on sign-swap permutation.

Multi-task learning has attracted substantial interest over recent years and a large volume of work exists within the machine learning literature (reviewed in Pan and Yang (2010)). There are many different approaches to MTL, including neural networks (Caruana, 1997), Bayesian approaches (Bakker and Heskes, 2003) including Gaussian processes (Bonilla et al., 2008; Boyle and Frean, 2005), kernel methods (Evgeniou et al., 2005), collaborative filtering (Abernethy et al., 2009) and learning a set of features shared between the tasks (Argyriou et al., 2007). Multi-task learning using GP models has also received a lot of attention in the spatial statistics field, where it is referred to as “co-kriging” (Cressie, 1993). In spite of this rich literature, to date there are only a handful of applications of MTL to neuroimaging: Zhang and Shen (2012) presented an approach to combine different imaging modalities to predict multiple clinical variables from regionally averaged structural MRI data. This approach induced coupling between the tasks at the feature selection stage, the PR models generating the final predictions were learned independently. Zhou et al. (2013) presented a multi-task regression approach to predict cognitive decline longitudinally, based on a set of regionally averaged cortical surface features and used structured regularisation penalties to induce coupling between the tasks. Varoquaux et al. (2010) presented an application of MTL to the estimation of functional connectivity matrices from fMRI data and Leen et al. (2011) presented a method to discriminate somatosensory stimuli based on a set of independent component analysis factor loadings derived from fMRI data. The work of Leen et al. (2011) shares similarities with the approach presented here in that is a GP model, but it differs in that it is asymmetric, whereby information is only shared in a uni-directional manner between tasks, and therefore has a different application focus to the present work (see Discussion). Also in contrast to these previous applications, scalability to voxel-wise analysis was an important motivating factor behind the present work because such analyses are important for exploratory neuroimaging data analysis.

In view of the related work outlined above, the contributions of this paper are the following: (i) a translational application of a symmetric MTL approach to whole-brain (voxel-wise) neuroimaging data; (ii) a comparison of the derived MTL models with conventional decoding approaches that learn each task independently (“single-task learning”/STL); (iii) contribution of a method for transforming MTL regression models into classification models, which is important because classification models are far more common in decoding studies; (iv) a comparison of free-form and restricted covariance structures for modelling inter-task dependencies and (v) presentation and evaluation of a brain mapping method for mapping discriminating brain regions in an MTL context. We evaluated all MTL and STL methods for predicting a range of contrasts from a publicly available dataset and hypothesized that MTL would lead to improved accuracy relative to STL and that the patterns of predictive weights would be more consistent across subjects owing to the coupling induced by the model.

## Methods

### Multi-task learning using Gaussian processes

In this section, we describe the Gaussian process MTL (GP-MTL) approach employed here, which is based on the approach outlined in (Bonilla et al., 2008). Further background on GP models for regression and classification can also be found in Rasmussen and Williams (2006). For didactic purposes, we describe GP-MTL for regression first, then generalise to classification. We also provide a simple simulation in the supplementary material illustrating the concepts introduced in the next few sections. We begin with a dataset {**X**, **y**}, where $X={\left[x_{1},\ldots ,x_{n_{x}}\right]}^{T}$ is an *n*_*x*_ × *d* matrix containing *d*-dimensional data vectors. In the most general case, **y** is an *n*_y_ × 1 vector of target variables that are grouped into *m* tasks. The goal is then to learn a set of *m* functions that predict the data as accurately as possible. We refer to the case where *n*_y_ = *mn*_*x*_ as a complete design, in that each input has an associated target value for every task. Multi-output models with no missing data provide an example of such a case. In many other cases, however, the design is incomplete. An important special case of an incomplete design is one where each input is associated with only one output and the tasks are coupled through the inputs. The fMRI dataset evaluated here provides an example of a scenario where such an incomplete MTL design would be appropriate. Clearly, STL is also a special case of MTL with *m* = 1.

For multi-task regression, we model the real-valued targets using a likelihood with *m* latent functions, collectively referred to as **f** = [**f**_1_^*T*^,…,**f**_*m*_^*T*^]^*T*^. Each function, **f***_q_*, has an associated Gaussian noise term *σ*_*q*_^2^. We apply a zero mean GP prior to the latent functions, *p*(**f**|**X**,***κ***) ∼ *N*(**f**|**0**,**K**) with a covariance function (i.e. kernel) given by:

(1) $$k\left(\left(x,p\right),\left(z,q\right)\right)=K_{\mathrm{pq}}^{f}k^{x}\left(x,z\right).$$

Here *K*_*pq*_^*f*^ denotes the covariance between task *p* and *q*, *k*^*x*^(**x**,**z**) describes the covariance between input data points and **K** is an *n_y_* × *n_y_* matrix evaluating the covariance function at all data points. The input covariance is typically taken as one of the covariance kernels used in a conventional STL framework (e.g. linear covariance or squared exponential/Gaussian covariance). We use ***κ*** to denote any hyperparameters on which the covariance function depends and collect the noise variables in a vector ***σ*** = [*σ*_1_,…,*σ*_*m*_]^*T*^ ***σ***^*2*^ = [*σ*^*2*^_1_,…,*σ*^*2*^_*m*_]^*T*^. Inference then proceeds by computing the posterior distribution of the latent function by Bayes rule:

(2) $$p\left(f|X,y,\sigma ,\kappa \right)=\frac{p\left(y|f,\sigma \right)p\left(f|X,\kappa \right)}{p\left(y\left|X,\sigma ,\kappa \right)\right)}.$$

Details about how to compute this distribution depend on the particular form used for the likelihood, *p*(**y**|**f**,***σ***), and will be discussed later (see also Rasmussen and Williams (2006)).

### 2.2 Multi-task learning with complete designs: multi-output regression

Before considering the most general case of MTL, we first consider the special case of a complete design. To simplify things further, we consider multi-output regression, where: (i) the outputs are continuous under the Gaussian noise model given above and (ii) the input data points are the same for each of the different outputs. In other words, each unique input vector is associated with a set of *m* targets. For notational convenience we also assume that the data samples are ordered such that the samples belonging to each task are clustered in blocks. In this case, the (noise-free) covariance function assumes a block diagonal structure and can be efficiently represented using a Kronecker product:

(3) $$K=K^{f}⊗K^{x}.$$

Here **K***^f^* is an *m* × *m* positive definite matrix describing the covariance between tasks and **K***^x^* describes the covariance between each (unique) data point. We can then rewrite Eq. (2) as:

(4) $$p\left(f|X,y,\sigma ,\kappa \right)=\frac{N\left(y|f,D⊗I_{n_{x}}\right)N\left(f|0,K\right)}{N\left(y|0,\Sigma \right)}$$

where **D** is a diagonal noise matrix with ***σ^2^*** along the leading diagonal, and

(5) $$\Sigma =K+D⊗I_{n_{x}}.$$

Note that in a multi-output regression model under Gaussian noise, the maximum likelihood estimates of the regression coefficients are equal to those derived by estimating the regression coefficients for each task independently (see Bishop (2006)). In contrast, under the proposed model, the tasks are coupled a posteriori through the GP prior. We provide a simulation illustrating the coupling between outputs in a multi-output model in the supplementary material.

### Covariance function and parameter optimisation

The covariance function is the crucial element imbuing GP models with modelling flexibility and expressive power. In the case of MTL, it is responsible for coupling the samples belonging to each task. The covariance function is typically dependent on a set of parameters (***κ***), which in this case are the task covariance (**K***^f^*) and any parameters relating to the input covariance (e.g. length scale parameters). In addition, any hyperparameters for the likelihood (e.g. ***σ***) must also be optimised to compute the posterior distribution of the latent function. Following the common notational convention, we collect all model parameters into a vector ***θ***. In the context of GP models, all these parameters can be efficiently optimised by maximising the log marginal likelihood (also referred to as “type II maximum likelihood”). In this work we use Carl Rasmussen's conjugate gradient optimiser (minimize.m, available from www.gaussianprocess.org/gpml/code) for finding optimal parameter values. For regression models, the log marginal likelihood is Gaussian:

(6) $$\log\left(p(y|X,\theta )\right)=-\frac{1}{2}y^{T}\Sigma^{-1}y-\frac{1}{2}\log|\Sigma |-\frac{n_{y}}{2}\log\left(2\pi \right).$$

Its partial derivatives, required for optimisation by gradient descent, can be computed using standard approaches (see Rasmussen and Williams (2006)) and are given by:

(7) $$\frac{\partial \log\left(p\left(y|X,\theta \right)\right)}{\partial \theta_{j}}=\frac{1}{2}\mathrm{tr}\left(\left({\alpha \alpha }^{T}-\Sigma^{-1}\right)\frac{\partial \Sigma }{\partial \theta_{j}}\right)$$

where we have defined a vector of weights ***α*** = **Σ**^− 1^**y**, which we will discuss in detail later.In this work, we use a simple linear covariance for the data points, **K**^*x*^ = **XX**^*T*^, having no hyperparameters. We employ two approaches to modelling the task covariance: (i) a free-form covariance matrix having *m*(*m* + 1)/2 distinct hyperparameters and (ii) a restricted covariance matrix having only one hyperparameter.

To estimate a free-form covariance matrix, it is necessary to constrain the matrix to be positive definite. This can be achieved by reparameterising using a Cholesky decomposition, $K^{f}=L^{f}L^{f^{T}}$. We let ***λ*** denote a vector containing the lower triangular (i.e. non-zero) elements of **L***^f^*, the entries of which are unconstrained and can be optimised safely. Similarly, to constrain the noise variables to be positive, we optimise them in the log domain. Thus, the final vector of hyperparameters we need to estimate for the free-form covariance is:

$$\theta ={\left[\kappa^{T},\log\left(\sigma^{T}\right)\right]}^{T}={\left[\lambda^{T},\log\left(\sigma_{1}\right),\ldots ,\log\left(\sigma_{m}\right)\right]}^{T}.$$

To compute the derivatives of the log marginal likelihood in Eq. (7), we require the derivatives of Eq. (5) with respect to the hyperparameters, which are:

(8) $$\frac{\partial \Sigma }{\partial L_{\mathrm{pq}}^{f}}=J_{\mathrm{pq}}L^{T}⊗K^{x}+L{\left(J_{\mathrm{pq}}\right)}^{T}⊗K^{x}$$

and

(9) $$\frac{\partial \Sigma }{\partial \log\left(\sigma_{q}\right)}=2\sigma_{q}^{2}J_{\mathrm{qq}}⊗I_{n_{x}}$$

where **J***_pq_* is an *m* × *m* indicator matrix equal to one in the *p*-th row and *q*-th column and zero elsewhere and $I_{n_{x}}$ is an *n_x_*-dimensional identity matrix.

For the restricted covariance matrix, we use a parametric form, given by:

(10) $$K^{f}=\left(1-\gamma \right)1_{m\times m}+m\gamma I_{m}$$

where **1**_*m* × *m*_ is a matrix of ones. This form for the covariance matrix is a GP equivalent of a method presented in (Evgeniou et al., 2005), and has the effect of forcing the predictive weights for each task to be similar to their common average, with the parameter *γ* ∈ [0,1] governing the strength of the coupling. Many other forms for the task covariance are possible, which enables prior knowledge about possible relationships between the tasks to be encoded (e.g. Sheldon (2008)). Since *γ* is constrained to the unit interval, we apply a logit variable transformation before optimisation. Thus, the final vector of hyperparameters we need to optimise for the restricted task covariance is:

$$\theta ={\left[\mathrm{logit}\left(\gamma \right),\log\left(\sigma_{1}\right),\ldots ,\log\left(\sigma_{m}\right)\right]}^{T}.$$

In addition to the derivatives of the noise parameters given above, we require derivatives of the covariance with respect to the hyperparameter *γ*, which are:

(11) $$\frac{\partial \Sigma }{\partial \mathrm{logit}\left(\gamma \right)}=\gamma \left(1-\gamma \right)\left[mI_{m\times m}-1_{m\times m}\right]⊗K^{x}.$$

### Generalising multi-task learning to incomplete designs

The foregoing description assumed a complete design (i.e. that the covariance assumes a Kronecker product structure). The covariance function specified in Eq. (1) is also suitable for incomplete case where: (i) the inputs may be distinct for each output or (ii) the number of outputs are not the same for each input. However, this requires some modification to the notation. One possible approach is to employ a structured “communication matrix” to handle missing data as in Skolidis and Sanguinetti (2011). In this work we pursue an alternative approach based on the element-wise (Hadamard) product. To keep the notation simple, we assume for the remainder of this paper that *n*_*x*_ = *n*_*y*_ = *n* and use *n* to refer to the total number of samples in the dataset. Note that this does not entail a loss of generality because the inputs can be duplicated as is implicitly done by the Kronecker product. We define an *n* × *m* indicator matrix **M** representing task membership, where *M*_*ip*_ = 1 if sample *i* belongs to task *p* and zero otherwise. Using this notation, we write: **K**^*F*^ = **MK**^*f*^**M**^*T*^ and denote the noise free covariance function by **K** = **K**^*F*^ ⊙ **K**^*X*^. Here, ⊙ denotes the element-wise product and the (*n* × *n*) matrix **K***^X^* denotes the covariance between all data points. Note that this is distinct from **K***^x^*, which describes the covariance between data points shared between outputs in a complete design. **K***^X^* can be thought of as the input covariance component of the full Kronecker product with missing data removed. We add noise by **Σ** = **K** + **N**, where **N** is a diagonal noise matrix with **M*σ*** on the leading diagonal. Using this notation, the derivatives of the covariance function with respect to the hyperparameters can be derived in the obvious way, equivalently to Eqs. (8), (9) and (11). For example,

$$\frac{\partial \Sigma }{\partial L_{\mathrm{pq}}}=M\left(J_{\mathrm{pq}}{\left(L^{f}\right)}^{T}+L^{f}{\left(J_{\mathrm{pq}}\right)}^{T}\right)M^{T}⊙K^{X}.$$

### Making predictions in regression models

For multi-task regression, the Gaussian likelihood leads to a Gaussian posterior in Eq. (2). Thus, the standard closed form equations for GP prediction apply (Rasmussen and Williams, 2006), and we can write the predictive distribution for a test point **x**_∗_ from task *q* as:

(12) $$p\left(y_{*}|X,y,x_{*},\theta ,q\right)=N\left(y_{*}|\mu_{*},\sigma_{*}^{2}\right)$$

(13) $$\mu_{*}={\left(k_{q}^{F}⊙k_{*}^{X}\right)}^{T}\alpha$$

(14) $$\sigma_{*}^{2}=k_{**}^{F}k_{**}^{X}-{\left(k_{q}^{F}⊙k_{*}^{X}\right)}^{T}\Sigma^{-1}\left(k_{q}^{F}⊙k_{*}^{X}\right).$$

Here, **k**_*q*_^*F*^ denotes the *q*-th column of **K***^F^*, **k**_∗_^*X*^ denotes the input covariance of the test point and *k*^*F*^_∗ ∗_ and *k*^*X*^_∗ ∗_ refer respectively to the task and input variances of the test point.

### Turning regression models into classifiers

Most applications of MTL in a GP context aim to solve regression problems (e.g. Boyle and Frean (2005); Alvarez and Lawrence (2008); Bonilla et al. (2008)). However, in neuroimaging studies, applications of classification vastly outnumber applications of regression. Therefore, we propose a straightforward approach for generalising GP-MTL models to classification. We employ a sigmoidal likelihood function to model the class labels then compute a Gaussian approximation the posterior distribution. To achieve this, we first replace the Gaussian likelihood in Eq. (4), with a cumulative Gaussian or probit likelihood, *p*(*y*_*i*_|*f*_*i*_) = *Φ*(*y*_*i*_*f*_*i*_), where *Φ*(*z*) = *∫* _−*∞*_^*z*^N(*x*|0, 1)*dx*, to model the binary class labels *y*_*i*_ ∈ {− 1, 1}:

(15) $$p\left(f|X,y,\kappa \right)=\prod_{i=1}^{n}\;p\left(y_{i}|f_{i}\right)\frac{N\left(f|0,K\right)}{p\left(y|X,\kappa \right)}.$$

In this case, the nonlinear likelihood means that neither the posterior nor the marginal likelihood admit closed form solutions, so we approximate both with the expectation propagation (EP) algorithm (Minka, 2001). Expectation propagation is well-known to provide highly accurate estimates of the posterior distribution and marginal likelihood (Nickisch and Rasmussen, 2008) and is the approximation of choice for binary GP classification. In preliminary work, we also found EP to provide more accurate predictions than an alternative approach based on optimal scoring (Hastie et al., 1993, 1995), which has recently been applied to neuroimaging (Grosenick et al., 2008, 2013). We refer the reader to (Rasmussen and Williams, 2006) for further details about EP.

### Computing predictive weights in the input space

There are two equivalent perspectives on GP models, the weight- and function space views (Rasmussen and Williams, 2006). For the foregoing description, we adopted the function space view because for high-dimensional data with relatively few samples, the predictive equations are more efficient and non-linear relationships can be modelled. However, it is also highly desirable to visualise the discriminating patterns in the input (i.e. voxel) space, which can be achieved by adopting the weight space view. This will require introducing some additional notation: we let $\tilde{X}$ represent the *n* × *md* block diagonal matrix obtained by stacking the elements of **X** belonging to each task in a block-wise fashion. We let **w** = [**w**_1_^*T*^,…,**w**_*m*_^*T*^]^*T*^, where each **w***_q_* is a *d* dimensional vector of predictive weights for task *q*. In correspondence with the function space view, the prior over the predictive weights is Gaussian, *p*(**w**|***κ***) ∼ *N*(**w**|0,**Σ**^*p*^). Following the approach described in our previous work (Marquand et al., 2010), we write $K=E\left[{\mathrm{yy}}^{T}\right]=\tilde{X}E{\mathrm{ww}}^{T}{\tilde{X}}^{T}$ and see that the covariance function used here corresponds to a prior over the weights where **Σ**^*p*^ is a *dm* × *dm* Toeplitz matrix constructed by stacking diagonal submatrices such that the *j*,*k*-th block has $\frac{1}{d}K_{j,k}^{f}$ along the leading diagonal. The off-diagonal components of this prior induce coupling between the tasks. The posterior over the weights is then:

(16) $$p\left(w|\tilde{X},y,\theta \right)=N\left(y|\hat{w},S\right)$$

(17) $$\hat{w}={\left({\tilde{X}}^{T}N^{-1}\tilde{X}+{\left(\Sigma^{p}\right)}^{-1}\right)}^{-1}{\tilde{X}}^{T}N^{-1}y$$

(18) $$S={\left({\tilde{X}}^{T}N^{-1}\tilde{X}+{\left(\Sigma^{p}\right)}^{-1}\right)}^{-1}$$

From this it follows that the equations for predicting an unseen data point from task *q* are simply:

(19) $$p\left(y_{*}|\tilde{X},y,x_{*},\theta ,q\right)=N\left(y_{*}|\mu_{*},\sigma_{*}^{2}\right)$$

(20) $$\mu_{*}=x_{*}^{T}{\hat{w}}_{q}$$

(21) $$\sigma_{*}^{2}=x_{*}^{T}S_{q,q}x_{*}$$

where ${\hat{w}}_{q}$ is the weight vector for task *q* and **S**_*q*,*q*_ is the corresponding block of the posterior covariance matrix. In practice, computation of Eq. (17) is infeasible for high-dimensional data because it involves the inversion of a large dense matrix $\left({\tilde{X}}^{T}N^{-1}\tilde{X}+{\left(\Sigma^{p}\right)}^{-1}\right)$, but this can be easily overcome using the one of the Woodbury identities, i.e.:

(22) $$\hat{w}=\Sigma^{p}{\tilde{X}}^{T}{\left(\tilde{X}\Sigma^{p}{\tilde{X}}^{T}+N\right)}^{-1}y=\Sigma^{p}{\tilde{X}}^{T}\alpha .$$

We note in passing that an equivalent trick can be employed to compute the posterior variance if the dimensionality is low enough that the *md* × *md* covariance matrix can be stored in computer memory. This could be very useful, for example to derive marginal variances for the weights at each voxel, but is impractical here because *md* is in the order of 600,000 (≈35,000  voxels × 18 subjects).

From the two sets of predictive equations (Eqs. (13) and (20)), it is clear that ***α*** and $\hat{w}$ play equivalent roles: the inner product between either quantity and the data determines the mean of the predictive distribution (in the feature or input space respectively). A similar correspondence exists for classification and vectors of predictive weights directly analogous to ***α*** and $\hat{w}$ can be easily derived to describe the mean of the Gaussian approximation to the posterior distribution. See Rasmussen and Williams (2006) for more details. For didactic purposes we show a simple simulation of the effects of MTL on the predictive weights in the supplementary material.

The predictive weights for the comparison STL models (denoted here by ${\hat{w}}^{s}$) can be computed using the approach described in our previous work (Marquand et al., 2010). To enable a fair comparison of the proposed MTL models with the STL models for which data are pooled across subjects (see below), it will also be useful to decompose the weight vectors for pooled models into the components attributable to each subject. These subject-specific weight vector components can easily be extracted owing to the linearity of the predictive weights. In other words, because a (*d* × 1) STL weight vector can be written as ${\hat{w}}^{s}=\sum_{q=1}^{m}\;{\hat{w}}_{q}^{s}$, where *q* = 1,…,*m* indexes subjects in the pooled STL model.

### Visualising the discriminating pattern through predictive mapping

The most common measure employed in neuroimaging for mapping the discriminative pattern is a spatial representation of the weight vector ($\hat{w}$ in a GP context). However, if we are interested in inferring the contribution of each brain region to the prediction, the weights only provide part of the story. As is clear from Eqs. (13) and (20), we cannot ignore the contribution of the data. In some cases, this has been tackled by presenting both weight maps and t-statistic images and considering both in drawing conclusions from the data (e.g. Mourao-Miranda et al. (2005); Marquand et al. (2012)).

In this work, we use the weight vector for assessing the reproducibility of the spatial patterns (because this is principally a property of the model) but we propose an alternative approach for performing inference over the discriminative brain regions. For a given test sample, (**x**_∗_)_*i*_, the predictive mean for task *q* is given by ${\left(\mu_{*}\right)}_{i}={\left(x_{*}\right)}_{i}^{T}{\hat{w}}_{q}=\sum_{j=1}^{d}\;{\left(x_{*}\right)}_{\mathrm{ij}}{\hat{w}}_{j}$, where (**x**_∗_)_*ij*_ denotes the *j*-th voxel in the *i*-th sample. This suggests a natural approach for mapping the total contribution of each voxel to classification: First, for every test sample, we compute:

(23) $${\left(x_{*}\right)}_{i}⊙{\hat{w}}_{q}.$$

Note that the predictive mean can be recovered by simply summing over this quantity. Assuming an appropriate cross-validation approach is employed, this yields *n* images – one for each test sample – which can then be summarised using an appropriate statistical testing procedure. In this work, we use a one-sample *t*-test (against zero). We then threshold this image using permutation testing as described in the next section. Finally, this thresholded image can be mapped across all voxels in the same way as the weight vector. We refer to this procedure as “predictive mapping” to distinguish it from the “discriminative mapping” approach commonly used in neuroimaging (i.e. mapping the weight vector).

Adopting the predictive mapping approach provides two advantages: first, it is intuitively appealing in that it quantifies the total contribution of each brain region to making the predictions we are actually assessing, not just the contribution of the weights. Second, it allows us to take a statistical view of the behaviour of the classifier within the test (or training) set. In contrast, the weight vector effectively provides a point estimate, which becomes problematic in a cross-validation context, where a distinct weight vector is estimated for each fold and must be summarised in some way. This has been done in neuroimaging studies by: (i) presenting a single example weight vector (e.g. from one cross-validation fold); (ii) presenting a single weight vector image derived after retraining the model with all data or (iii) averaging the weights over all cross-validation folds. None of these alternatives faithfully represent the actual behaviour of the classifier: the first alternative provides only a single estimate, which may be misrepresentative if the weights are not highly reproducible; the second alternative suffers from a similar problem and presents a weight vector that was not used for making any of the predictions; the third alternative involves an averaging process, which may result in artificially smoothing the weight maps.

### Permutation test to identify discriminating regions

To highlight the most important regions of the discriminating pattern across all samples, and to facilitate comparison of the different methods, we threshold the predictive maps using a sign-swap permutation procedure. Similar permutation testing approaches are commonplace in neuroimaging both for mass-univariate analysis (Meriaux et al., 2006; Nichols and Holmes, 2002) and pattern recognition (Brodersen et al., 2012; Mourao-Miranda et al., 2005; Valente et al., in press; Wang et al., 2007). We emphasize that we adopt this approach to identify the most important regions in the discriminative patterns, thereby assisting their interpretation. We do not suggest that these are the only regions that are important because GP models, like other kernel methods, are characterised by a non-zero contribution from every brain region.

To achieve this, we first compute predictive maps for every subject as described above, yielding a t-statistic for each voxel. We then construct a null distribution for this statistic by randomly permuting the images 1000 times. For each permutation, we multiply the sign of each image randomly by either +/− 1. By permuting entire images we accommodate the spatial correlation structure in the data. We then retrain the model and compute the permuted predictive map. Finally we derive a p-value for each voxel by counting the number of times the permuted statistic for that voxel exceeds the true statistic and dividing by 1000. For the present work we display maps thresholded at the arbitrary, but commonly used, value of p < 0.001.

### Evaluation dataset: overview

We evaluate the proposed MTL models on a publicly available dataset downloaded from the OpenfMRI repository (http://openfmri.org). The Open fMRI project is managed by Russ Poldrack at the University of Texas at Austin, with computing resources provided by the Texas Advanced Computing Center. It is funded by a grant from the National Science Foundation (OCI-1131441).

The data employed for the present work are described in detail in Uncapher et al. (2011). In brief, 18 healthy subjects (9 females, aged 18–27 years) were scanned on 10 occasions with a T2*-weighted gradient echo spiral-in/out imaging sequence while performing a Posner cueing paradigm task. For each of the 10 fMRI runs, 190 volumes were acquired on a 3T Signa MRI scanner (GE Medical Systems) with acquisition parameters: repetition time = 2 s; echo time = 30 ms; flip angle 75°; 64 × 64 matrix; 30 4 mm axial slices and 3.44 mm^2^ in-plane resolution. To assist accurate normalization of subjects to standard space, a high resolution T1-weighted spoiled gradient recalled structural image was also acquired for each subject. These images each had 130 1.5 mm thick slices, a 256 × 256 matrix and 0.86 mm^2^ in-plane resolution.

The Posner task investigated the effect of top-down versus bottom-up attentional processes on episodic memory encoding. During the task, subjects viewed two white boxes to the left and right of a central fixation crosshair. A green arrow cue presented for one second signaled the beginning of each trial. This arrow pointed either left or right, cueing subjects to covertly shift their attention to the box indicated. On 82% of trials, a line drawing of an object appeared in the cued box (“Valid trials”), and in the remainder of trials it appeared in the non-cued box (“Invalid trials”). Subjects were required to indicate by button press whether the drawing depicted a real object or an imaginary class of objects referred to as “greebles”. After the final scanning session, subjects performed a surprise memory test where they viewed a series of line drawings (350 studied and 180 unstudied items) and were asked to indicate whether each item was viewed during any of the scanning sessions, and indicate their level of confidence for that decision. This allowed the scanned stimuli to be categorised along a number of dimensions, such as whether they were successfully encoded (“hit” or “miss”), whether the cue was valid or invalid, what the confidence associated with the encoding was and whether a real object or a greeble was presented (Table 1). See Uncapher et al. (2011) for further experimental details.

### Evaluation dataset: neuroimaging data preprocessing

Neuroimaging data preprocessing was performed using the SPM12b software. Data from each fMRI run were first realigned to the mean image in each timeseries then coregistered to the T1-weighted structural image from that subject. These structural images were segmented and normalised to a standard space using the Segment tool in SPM12b (formerly “new segment”). The deformations obtained from the segmentation were then applied to normalise the fMRI images, during which they were resampled to the original acquisition resolution. These images were then smoothed with an isotropic 8 mm Gaussian kernel prior to analysis.

Since this is a relatively fast fMRI design, GLM regression coefficient images were used as samples to train the classification models. The GLM model design followed that reported in Uncapher et al. (2011), where an independent regressor was constructed for each experimental condition (Table 1) and convolved with the canonical haemodynamic response function provided by the SPM software. Movement parameters derived from the image realignment were also included as nuisance regressors. A high-pass filter cutoff of 128 s was specified for detrending with a cosine basis transform and the model was estimated using a classical least-squares approach. Note that an independent GLM model was estimated for each fMRI run. After model estimation, the resulting GLM coefficient images were masked to exclude non-brain tissue and supplied to the classifier for analysis. For comparison with the predictive maps from the classifiers, univariate statistical parametric maps (SPMs) were also generated using the following procedure: at the first level, one sample fixed effect t-contrasts were performed for each of the groups of regressors used to train the classifiers (described below). The resulting contrast images were then entered into a second-level flexible factorial random effects model where a two sample *t*-test was used to assess the difference between the conditions in a mass-univariate sense.

Clearly, this experiment has a complex design and there are many possible hypotheses that can be tested. To obtain an unbiased yet comprehensive estimate of the performance of the models evaluated, we adopted two analytical approaches. For the first approach we identified a priori three primary contrasts that are broadly representative of some of the most important experimental questions that the data could answer. We denote these by: (i) CUE v OBJ, corresponding to a task effect contrasting the activity patterns between cue and object presentation. This was constructed by contrasting regressors 1 and 2 with regressors 10 and 11 in Table 1; (ii) VAL vs INVAL, corresponding to the effect of cue type (valid or invalid) and is referred to in Uncapher et al. (2011) as a “bottom up attention effect” (regressors 10 and 11 with 13 and 14); (iii) HIT vs MISS, corresponding to successful encoding, that is, items that were subsequently remembered contrasted with those that were not (regressors 10 and 13 with 12 and 15). Since there are many other contrasts that may be of interest, we also followed a second approach where we trained binary classifiers to discriminate all pairwise combinations of regressors (66 in total).

### Classifier configuration and model assessment

For each of the contrasts noted above, we trained a total of four classification models. First, we trained simple baseline models where each subject was analysed independently (“single subject”). Next, we trained single classification models after pooling data from all subjects (“pooled”). These models are collectively representative of current practice in neuroimaging decoding studies. Next, we trained MTL learning models using the approach outlined above where task dependencies are modelled using free-form and restricted task covariance matrices (“MTL (F)” and “MTL (R)”). For each classifier, type-II maximum likelihood was used to estimate hyperparameters from the training set. To investigate the possibility of multiple modalities in the marginal likelihood, we performed several pilot runs using different starting points. For these data, this resulted in only small numerical differences to the predictions. In cases where evidence is found for multiple modes, a simple approach is to choose the hyperparameter settings having the largest value for the marginal likelihood.

We assessed the accuracy of each classifier using a cross-validation scheme where for each fold we excluded all data from one run (“leave-one-run-out cross-validation”). We also excluded data for the small number of runs that did not have at least one sample from each class. Note that in general classifiers were approximately balanced. Prior to classification, data were standardized across subjects within each fold using the mean and standard deviation from the training set. We report accuracy measures for all classifiers in addition to receiver operating characteristic (ROC) curves for the primary contrasts. For the single subject models, classification accuracies were averaged across subjects to provide an overall assessment of performance. To derive a summary ROC curve from the different single subject classifiers, we employed the simple method proposed in (Obuchowski, 2007).

### Generalisation to new subjects

The leave-one-run-out cross-validation approach described above provides an indication of the generalizability across runs within the same group of subjects. However, for many applications it is important to generalise to new subjects or tasks. For example, in a clinical setting predictions for new subjects are of primary interest. Surprisingly, the problem of transferring knowledge to new tasks that do not exist in the training set has received relatively little attention in the MTL literature and most applications employ validation schemes where partitions of data are withheld for all tasks.

To demonstrate the generalizability of the proposed method to new subjects, we evaluate the performance of MTL under a leave-one-subject-out cross-validation framework. This enables us to estimate generalizability to the population. For this purpose, we compare MTL (R) to a pooled model combining the data from all subjects. For MTL (F), generalisation to unseen data is more complex because it is necessary to estimate cross-covariances for tasks in the test set that do not exist in the training set. One potential solution to this problem was proposed in Skolidis and Sanguinetti (2012) and consists of constraining the magnitude of entries of the task covariance to the unit interval then employing a multinomial likelihood function to estimate the similarities between the tasks in the test and training sets. However, we do not pursue this approach here.

We also note that the proposed MTL method is equally well suited to other cross-validation approaches, subject to the constraints noted above and provided that the independence of training and test sets is preserved (see Pereira et al. (2009)).

### Pattern reproducibility

In addition to predictive accuracy, we compared classifiers based on the reproducibility of the patterns of predictive weights using Pearson product-moment correlation (i.e. cosine distance), both within and between subjects under leave-one-run-out cross-validation. This is a useful tool to quantify the coupling between the models that is induced by the MTL framework and can provide information about the variability of the patterns of responses between subjects. It is also important because a tradeoff exists between prediction accuracy and the reproducibility of spatial patterns under perturbations to the training set (Rasmussen et al., 2011; Strother et al., 2004). To date, this has meant that for some applications slightly less accurate models that show more reproducible patterns of weights may have been preferred. Multi-task learning potentially provides way to simultaneously achieve both objectives, providing models that are both accurate and reproducible.

While it may seem obvious that inducing coupling between tasks will result in weight vectors that are more similar to one another, it is important to point out that applying the proposed MTL framework does not necessarily lead to coupling between the weight vectors because the degree of task coupling is estimated from the data and can be estimated to be zero if the tasks are very different. Further, a high degree of coupling between tasks in one particular training set (i.e. cross-validation fold), does not imply that the weight vectors will be more reproducible across folds.

## Results

### Accuracy of illustrative contrasts

**Fig. 1:**
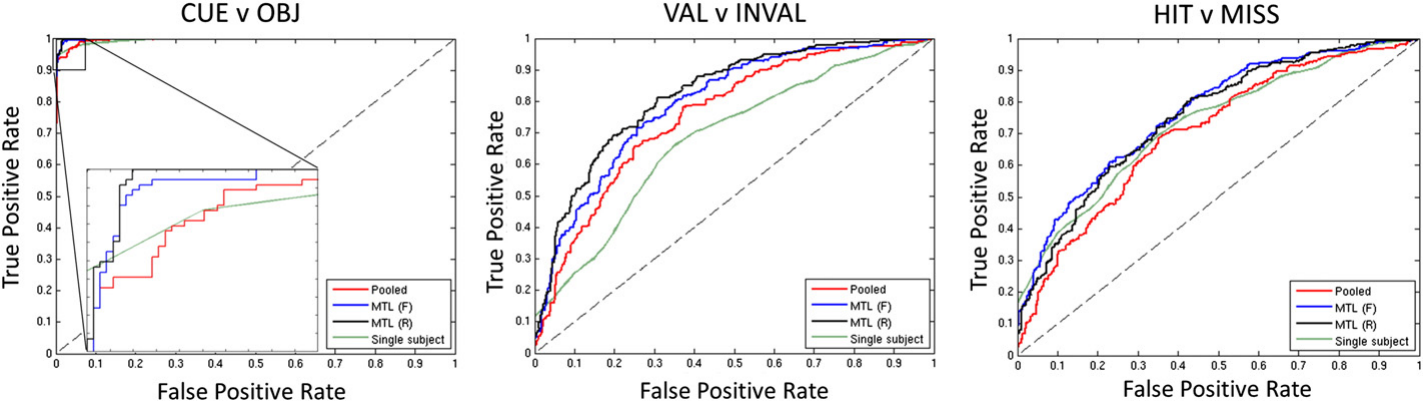
Receiver operating characteristic curves showing improved performance of MTL relative to STL approaches on the illustrative contrasts (leave-one-run-out cross-validation). For the CUE v OBJ contrast an enlargement is shown. Abbreviations: MTL (F) = multi-task learning with a free-form covariance, MTL (R) = multi-task learning with a restricted covariance, STL: single task learning.

The classification accuracies obtained on the primary contrasts (CUE v OBJ, VAL v INVAL and HIT v MISS) under leave-one-run-out cross-validation are summarised in Table 2. Receiver operating characteristic curves and the area under the curve (AUC) for each of these classifiers are presented in Fig. 1 and Table 3 respectively. These results indicate that for the illustrative contrasts, MTL lead to higher categorical classification accuracy than any of the other classifiers and also achieved better performance at nearly all decision thresholds. On these contrasts, MTL (F) and MTL (R) performed similarly.

### Accuracy of all pairwise contrasts

**Fig. 2:**
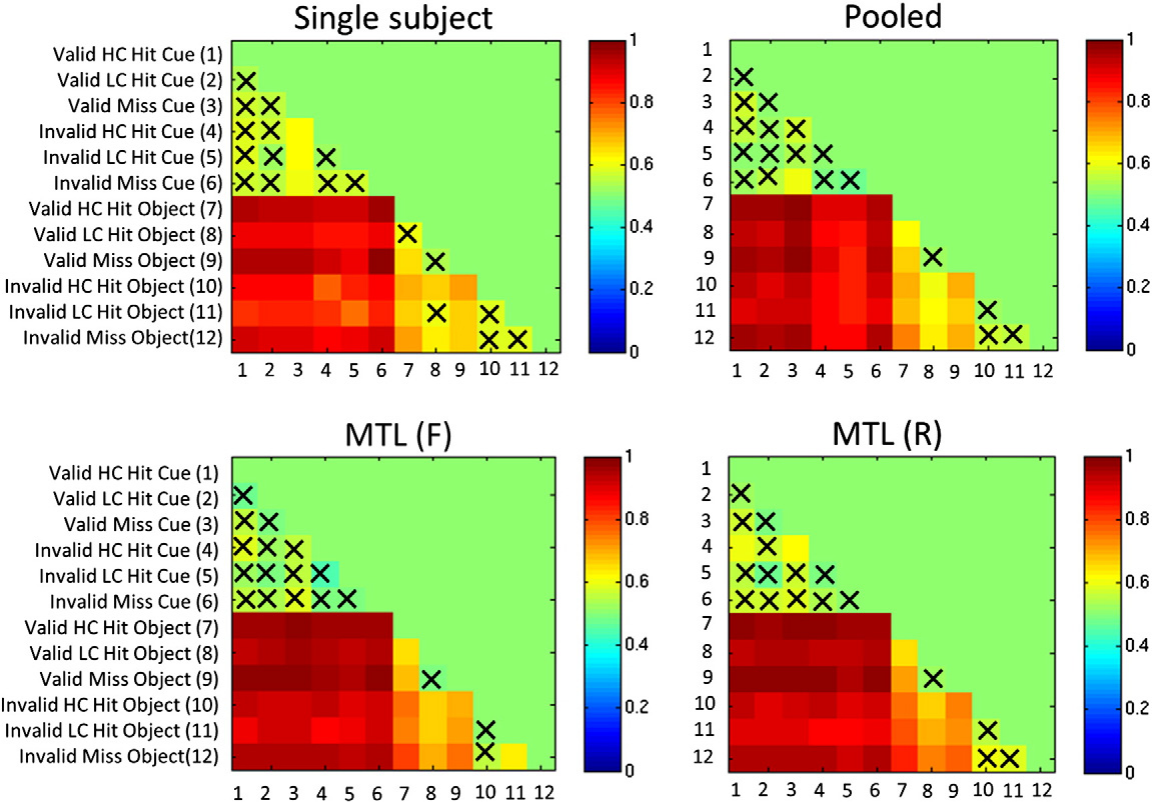
Accuracy of all classifiers for all pairwise contrasts (leave-one-run-out cross-validation). Crosses denote comparisons for which the classifier did not exceed 60% accuracy. Abbreviations: HC = high confidence, LC = low confidence, MTL (F) = multi-task learning with a free-form covariance, MTL (R) = multi-task learning with a restricted covariance.

Accuracies for each of the 66 pairwise contrasts under leave-one-run-out cross-validation are reported graphically in Fig. 2 and numerically in the supplementary material. To assist visualisation, the comparisons in Fig. 2 that were difficult to predict are indicated by crosses (i.e. classifiers that did not exceed 60% accuracy). These results indicate that: (i) it was more difficult to discriminate the Cue conditions from one another relative of any of the Object conditions from the Cue conditions or one another and (ii) MTL (F) and MTL (R) produced the highest performance over all pairwise constrasts with a slight advantage for MTL (R) over MTL (F).

**Fig. 3:**
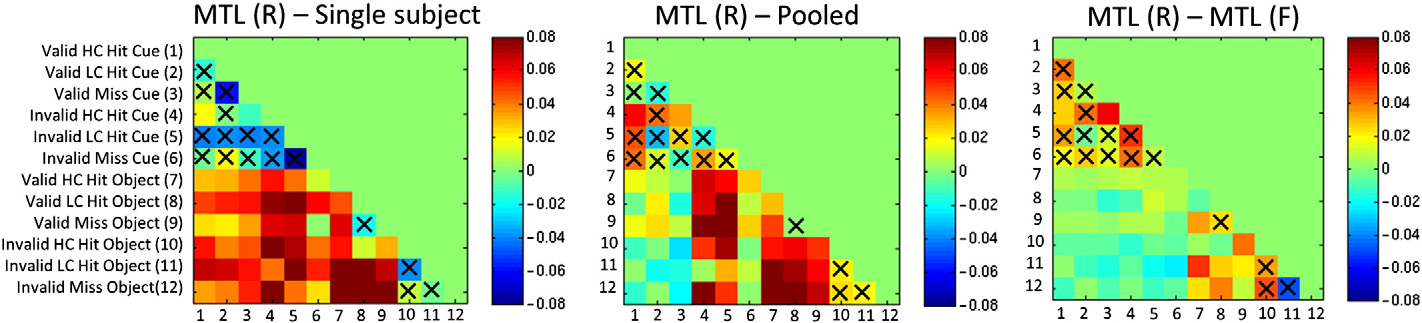
Accuracy of MTL (R) in relation to all other classifiers for all pair-wise contrasts (leave-one-run-out cross-validation). The colour scale denotes the difference in accuracy between MTL (R) and each comparison method. In this setting, MTL (R) leads to significantly improved performance relative to STL and MTL (F). Crosses denote comparisons for which the classifier did not exceed 60% accuracy. Abbreviations: HC = high confidence, LC = low confidence, MTL (F) = multi-task learning with a free-form covariance, MTL (R) = multi-task learning with a restricted covariance, STL = single task learning.

To further facilitate the comparison of the best-performing method, MTL (R), with the other methods, a graphical representation of the differences in accuracy is presented in Fig. 3 along with a high-level summary of the differences in Table 4. These comparisons illustrate that: (i) MTL (R) produced significantly higher accuracy than all other classifiers (Wilcoxon signed rank test); (ii) the difference in accuracy was greater for the contrasts that improved relative to the ones that did not and (iii) the contrasts where MTL (R) did not provide a performance improvement corresponded to contrasts that were the most difficult to predict and that in many cases could not be accurately predicted by any classifier. A similar pattern of results was observed for MTL (F) with respect to the other classifiers (Supplementary material). The pooled classifiers performed broadly similarly to the single subject classifiers.

To further facilitate the comparison of the best-performing method, MTL (R), with the other methods, a graphical representation of the differences in accuracy is presented in Fig. 3 along with a high-level summary of the differences in Table 4. These comparisons illustrate that: (i) MTL (R) produced significantly higher accuracy than all other classifiers (Wilcoxon signed rank test); (ii) the difference in accuracy was greater for the contrasts that improved relative to the ones that did not and (iii) the contrasts where MTL (R) did not provide a performance improvement corresponded to contrasts that were the most difficult to predict and that in many cases could not be accurately predicted by any classifier. A similar pattern of results was observed for MTL (F) with respect to the other classifiers (Supplementary material). The pooled classifiers performed broadly similarly to the single subject classifiers.

### Generalisation to new subjects

**Fig. 4:**
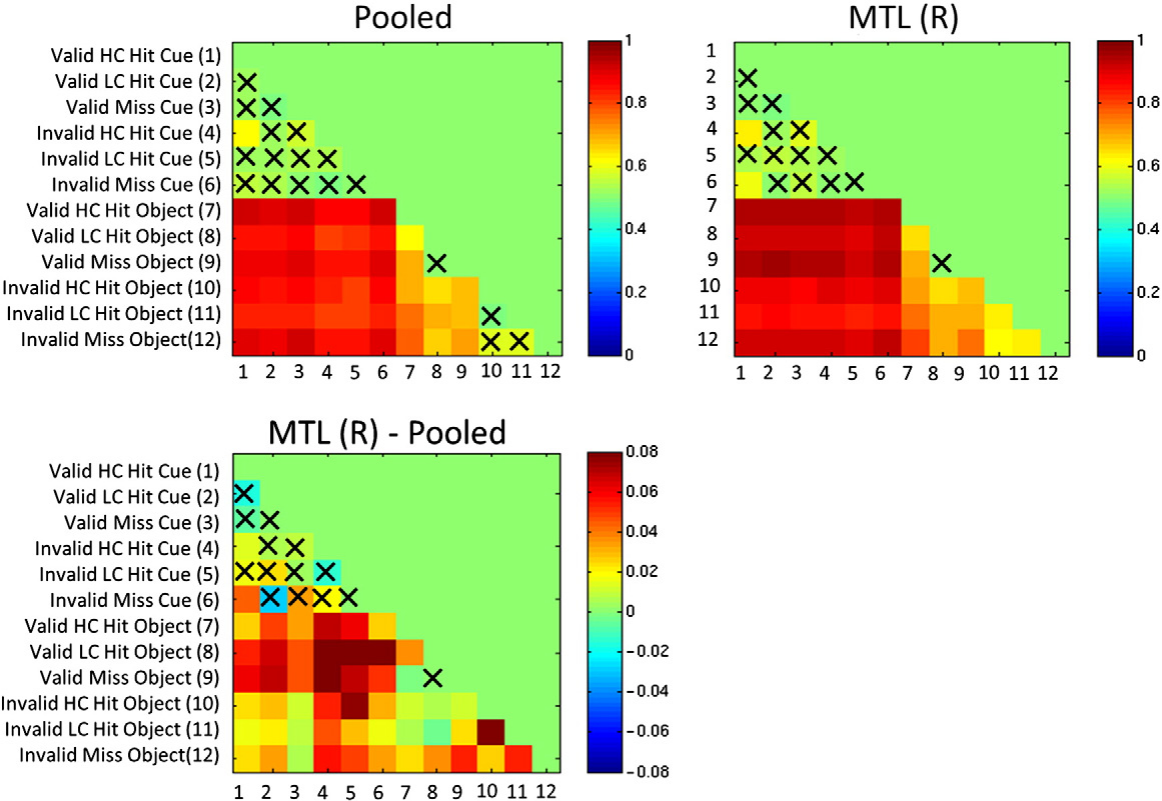
Top panels: Accuracy of MTL (R) and pooled classifiers for all pairwise contrasts (leave-one-subject-out cross-validation). Bottom panel: difference between MTL (R) and pooled classifiers (leave-one-subject-out cross-validation). In this setting, MTL (R) leads to significantly improved performance relative to pooled classifiers. Crosses denote comparisons for which the classifier did not exceed 60% accuracy. Abbreviations: HC = high confidence, LC = low confidence, MTL (R) = multi-task learning with a restricted covariance.

Consistent with the results reported above, under leave-one-subject cross-validation MTL (R) classifiers produced higher categorical classification accuracy than two of the three pooled classifiers and produced a greater AUC than all pooled classifiers (Tables 5 and 6). Accuracies for each of the 66 pairwise contrasts under leave-one-subject-out cross-validation are reported graphically in Fig. 4 and numerically in the supplementary material. For the pair-wise contrasts, MTL (R) produced higher classification accuracy for 89.39% of contrasts, with a mean increase of 3.81% accuracy (*p* = 1.1 × 10^− 7^, Wilcoxon signed rank test; Fig. 4). Conversely, the MTL (R) classifiers produced lower accuracy than the pooled classifiers for only 9.09% of contrasts with a mean decrease of (− 1.15%).

### Inter-task coupling

**Fig. 5:**
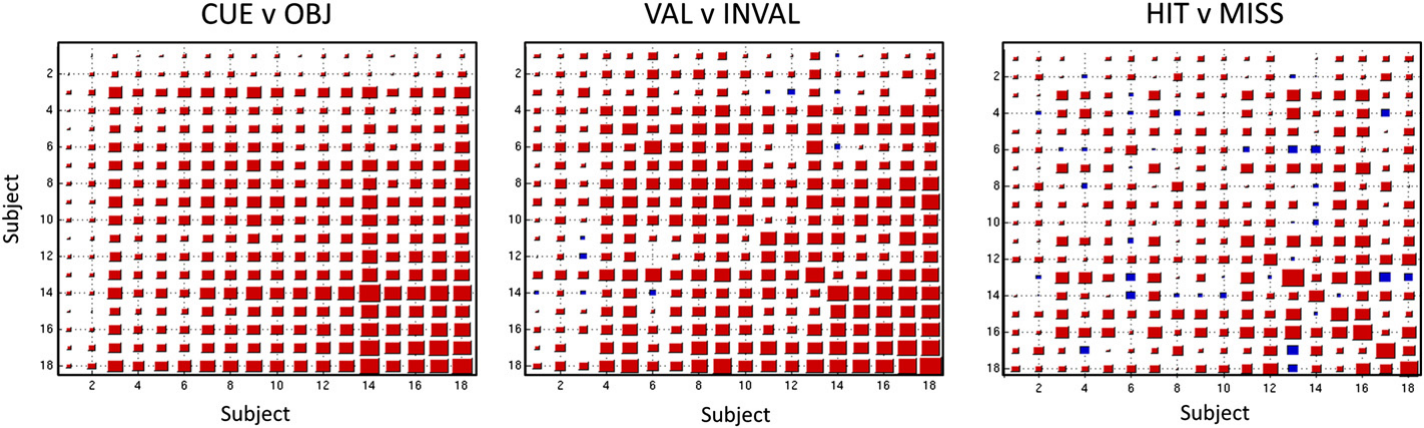
Hinton diagram showing examples of task covariance matrices for each of the primary contrasts. This illustrates the inter-task coupling learned by the MTL (F) classifiers. Shown are the covariances from the first leave-one-run-out cross-validation fold. Abbreviation: MTL (F) = multi-task learning with a free-form covariance.

In addition to the predictive accuracy, for many applications it is useful to quantify the coupling between tasks or subjects, which can be achieved by visualising the task covariance matrix (**K***^f^*). As illustrative examples, the task covariances for each of the primary contrasts for the MTL (F) classifier under leave-one-run-out cross-validation are provided in Fig. 5. These show that: (i) the overall coupling was strong for the CUE v OBJ and VAL v INVAL contrasts but weaker for the HIT v MISS contrast, which corresponds with the relative differences in classification accuracy afforded by MTL for each contrast; (ii) the first two subjects appear to be somewhat anomalous across all contrasts and are relatively weakly coupled to the other subjects.

### Reproducibility of the weight vectors

For brevity, only the results from the VAL v INVAL contrast are presented in detail. The weight vectors for the other contrasts (CUE v OBJ and HIT v MISS) show a similar behaviour, albeit with a slightly lower coupling induced for HIT v MISS. The reproducibility of the weight vectors for the VAL v INVAL contrast across leave-one-run-out cross-validation folds was high within subjects for the single subject, MTL (F) and MTL (R) classifiers (mean [SEM] correlation = 0.845 [0.028], 0.834 [0.012] and 0.905 [< 0.001] respectively). Reproducibility within subjects was substantially lower for the pooled classifier (0.702 [0.048]). Spatial representations for the weight vectors from each classifier are provided in the supplementary material.

**Fig. 6:**
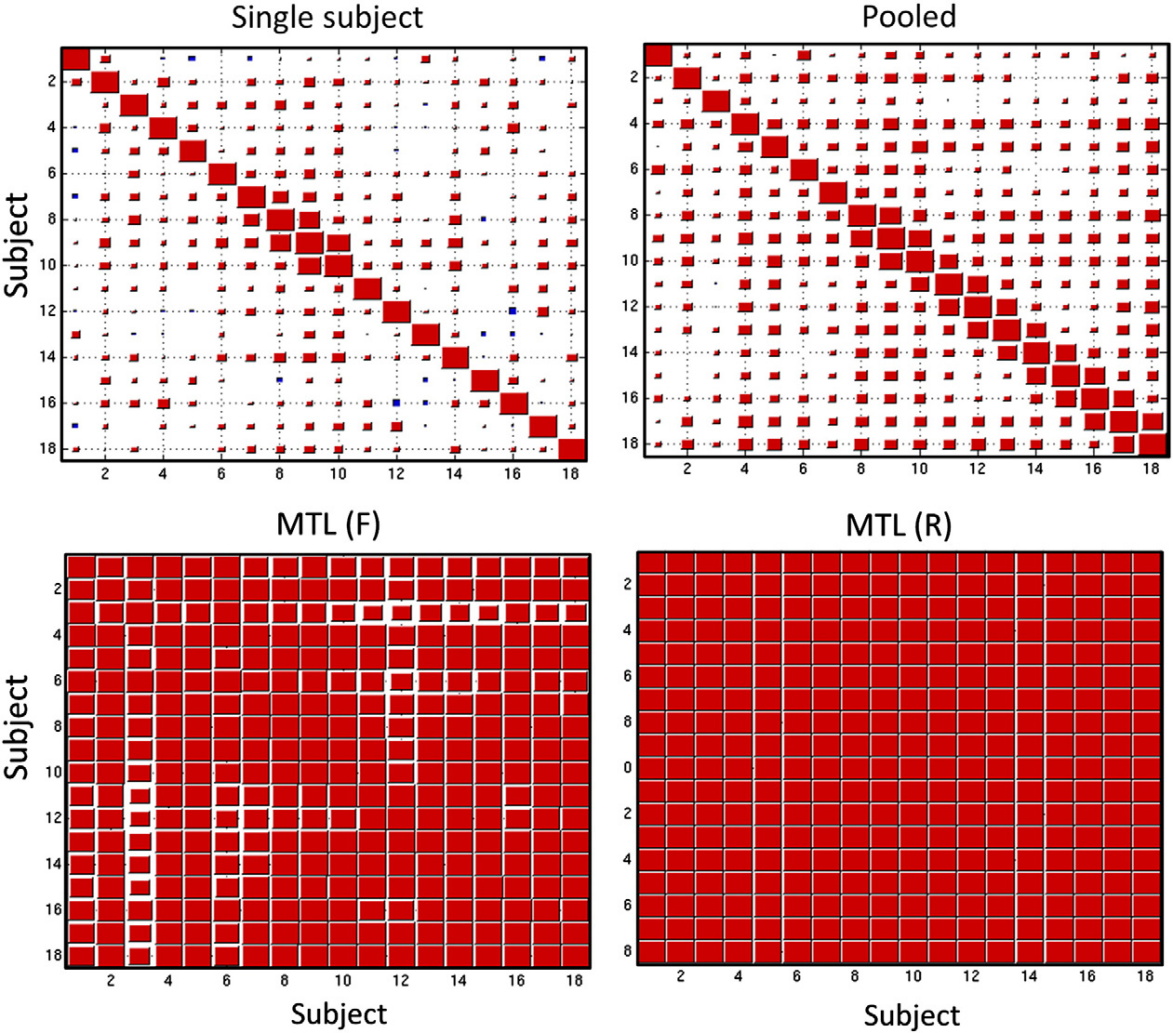
Hinton diagram showing the correlations between the weight vectors for each subject and classifier. This shows that both MTL approaches lead to more reproducible weight vectors. Abbreviation: MTL (F) = multi-task learning with a free-form covariance, MTL (R) = multi-task learning with a restricted covariance.

To assess the similarity of the weight vectors between different subjects for each classifier, the correlations between the mean weight vector for each subject are presented in Fig. 6. In contrast to the within-subject reproducibility, these results show that the weight vectors for different subjects from the single subject and pooled models are nearly uncorrelated. The differences between the weights for each subject are also apparent by inspection of the weight vectors themselves (see Supplementary material). Taken together, these results show that: (i) the single subject models were able to learn a reproducible set of weights across cross-validation folds but primarily learned idiosyncratic properties of each subject and (ii) the pooled models were less reproducible across cross-validation folds, and also seem to focus mostly on idiosyncratic properties of different subjects. In contrast, both MTL models learned a strong coupling between the tasks which enforced a high degree of similarity for the weight vectors, leading to high reproducibility between and within subjects. Note that the coupling between the weights was learned from the data and is not imposed directly by the MTL model. Also, the high reproducibility between the weight vectors of different tasks is not simply a result of including multiple subjects in the same model, because the between-subject reproducibility of the pooled models was low.

To assess the similarity of the weight vectors between different subjects for each classifier, the correlations between the mean weight vector for each subject are presented in Fig. 6. In contrast to the within-subject reproducibility, these results show that the weight vectors for different subjects from the single subject and pooled models are nearly uncorrelated. The differences between the weights for each subject are also apparent by inspection of the weight vectors themselves (see Supplementary material). Taken together, these results show that: (i) the single subject models were able to learn a reproducible set of weights across cross-validation folds but primarily learned idiosyncratic properties of each subject and (ii) the pooled models were less reproducible across cross-validation folds, and also seem to focus mostly on idiosyncratic properties of different subjects. In contrast, both MTL models learned a strong coupling between the tasks which enforced a high degree of similarity for the weight vectors, leading to high reproducibility between and within subjects. Note that the coupling between the weights was learned from the data and is not imposed directly by the MTL model. Also, the high reproducibility between the weight vectors of different tasks is not simply a result of including multiple subjects in the same model, because the between-subject reproducibility of the pooled models was low.

### Predictive maps

**Fig. 7:**
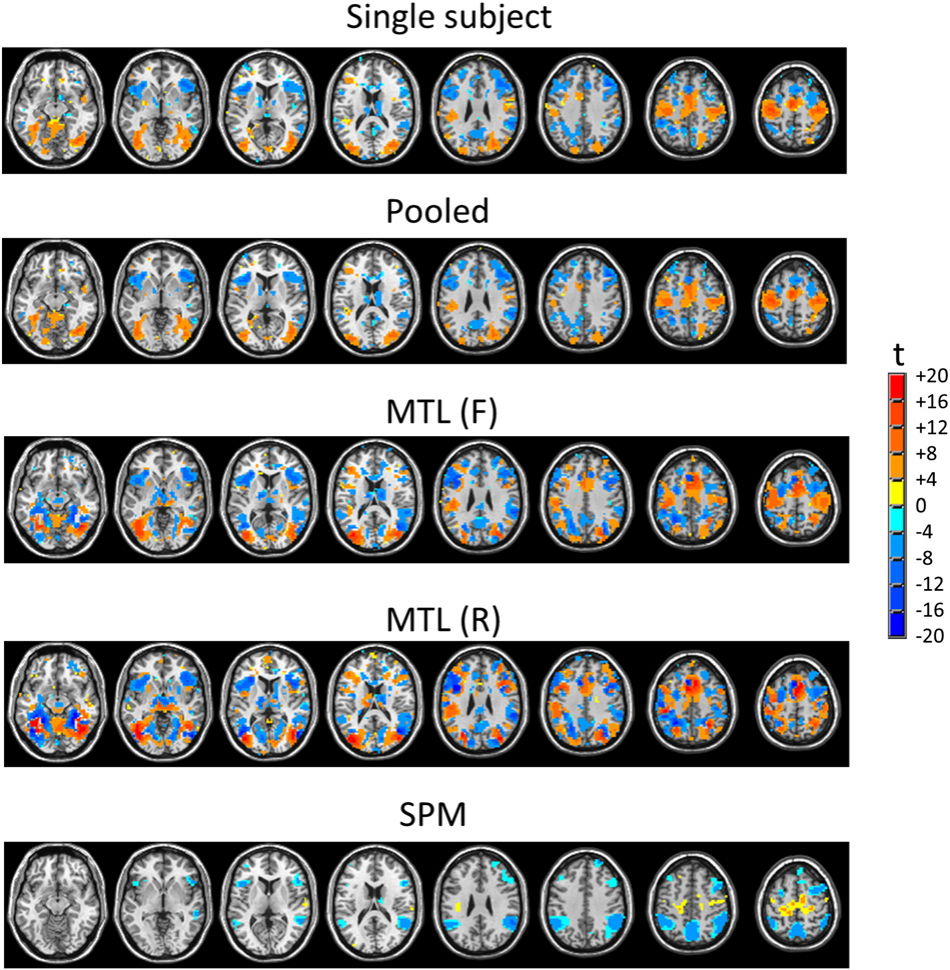
One sample *t*-test measuring the contribution of each brain region to the classifier prediction for the VAL v INVAL contrast. A mass-univariate SPM is also presented for comparison (see text for details). All maps were thresholded voxel-wise at p < 0.001. Abbreviation: MTL (F) = multi-task learning with a free form covariance, MTL (R) = multi-task learning with a restricted covariance, SPM = statistical parametric map.

Multivariate predictive maps derived from the one-sample *t*-tests are presented in Fig. 7 for the VAL v INVAL contrast (under leave-one-run-out cross-validation). For comparison with the predictive maps, mass-univariate SPMs for the same contrast are also presented. These maps have been thresholded at an uncorrected value of p < 0.001 to ensure a fair comparison with the predictive maps. All predictive maps indicate a similar pattern of effects across brain regions, but the MTL classifiers show more consistent effects in that the magnitude of t-statistics are higher and more voxels survive the p < 0.001 threshold, relative to the STL classifiers; from a total of 35,449 voxels in the brain mask, 11,536 survive thresholding in for MTL (R), relative to 9264 for MTL (F), 6662 for single subject classifiers and 5743 for pooled classifiers. All predictive maps show more voxels surviving thresholding relative to the SPMs (2704). The predictive maps and SPMs largely overlap, but it is notable that some of the regions showing strong univariate differences are not the most important for prediction (e.g. posterior cingulate cortex).

## Discussion

In this work, we demonstrated a translational application of MTL for fMRI data analysis. We evaluated several different MTL approaches based on GPs which enabled the automatic estimation of the covariance structure between fMRI data from different subjects. We compared the accuracy obtained by these models to commonly used STL approaches in predicting many contrasts in a publicly available fMRI dataset. In addition, we presented a novel method for mapping the predictive contribution of each brain region for MTL and STL models. We report four main findings: (i) combining tasks using MTL improved predictive performance for a majority of contrasts relative to single subject and pooled STL models; (ii) imposing a restricted covariance structure between tasks may improve performance if this structure is appropriate for the data; (iii) MTL models produced a more reproducible pattern of weights across subjects and cross-validation folds and (iv) were more effective for detecting brain regions predictive of the class labels relative to competing PR and mass-univariate models.

Multi-task learning is a natural paradigm for neuroimaging data analysis, and the approach pursued here – where each subject is framed as a task – is directly analogous to other types of problem to which MTL has classically been shown to be beneficial: for example, predicting subjective preferences for purchasing different products based on the preferences derived from other subjects (e.g. Evgeniou et al. (2005); Argyriou et al. (2007)) and predicting exam scores from students that are grouped into schools (e.g. Bakker and Heskes (2003); Evgeniou et al. (2005); Bonilla et al. (2008)). Such an approach can also be considered analogous to the use of mixed effects models in a mass-univariate context to model inter-subject variability in fMRI. Our results show that for neuroimaging data, this MTL approach leads to consistent improvements in predictive performance. For the dataset we considered, MTL provided the largest improvements over the single subject models (Table 4). This indicates that training an independent model for each subject makes inefficient use of the data available. This corresponds with results from the machine learning literature indicating that MTL is most beneficial for scenarios where each task has a small number of data points, but a large number of such tasks are available (Bakker and Heskes, 2003; Evgeniou et al., 2005; Sheldon, 2008). This is precisely the regime that is characteristic of many neuroimaging studies. In addition to improving predictive performance over the single subject models, MTL also consistently improved predictive performance over the pooled models (Table 4). This shows that the performance improvement afforded by MTL is not just due to learning from more data. Instead, it is important to model the relationships between scans to obtain optimal predictive accuracy.

In a neuroimaging context, the main focus for applications of MTL has been to predict multiple outputs based on a single structural MRI image for each subject (Zhang and Shen, 2012; Zhou et al., 2013), an approach also referred to as multi-output learning. As we have indicated, multi-output learning is a special case of MTL where the inputs are the same for each output. A related approach for accommodating multiple target variables in a multivariate linear regression was also recently proposed (Valente et al., in press), although this approach was not framed in an MTL context. One of the main contributions of this work is to demonstrate that MTL has a broader applicability for neuroimaging data analysis than has been demonstrated to date in that it is also suited to:(i) multi-subject fMRI studies and (ii) classification problems. In addition to the application demonstrated here, there are many other problems in neuroimaging data analysis for which MTL is a natural analytical approach. For example, for accommodating inter-scanner and inter-site variability, which is a major challenge in multi-site neuroimaging studies. Another notable difference between the present work and these previous applications is that it is framed within a kernel framework and therefore scales easily to the whole brain and can be used with non-linear covariance functions.

One of the only previous applications of MTL for fMRI data analysis of which we are aware presented an asymmetric MTL approach – also based on GPs – for discriminating somatosensory stimulation from auditory and visual stimulation (Leen et al., 2011). In asymmetric MTL, one task is designated as the primary task, and the others as secondary tasks. In contrast to the symmetric approach pursued in this paper, the goal is to improve the performance of the primary task, by using information from the secondary tasks. In other words, information is only allowed to flow from the secondary tasks to the primary task, and other dependencies (e.g. between secondary tasks) are ignored. Leen and colleagues reported that asymmetric MTL performed better than symmetric approaches. However, the MTL configuration employed was somewhat contrived in that the primary task was a somatosensory stimulation paradigm and the secondary tasks included somatosensory, visual and auditory stimulation. Further, for many fMRI tasks (such as the one considered here), it is not obvious which task should be designated as “primary”. While there is certainly a role for asymmetric approaches, symmetric MTL is better suited to routine neuroimaging data analysis.

An important feature of the GP approach we employed is the ability to specify or estimate a free-form covariance matrix to model relationships between the tasks (i.e. subjects). This provides useful information about the learned coupling between the tasks and confers MTL models with a high degree of flexibility; for example, it allows prior knowledge about the relationships between tasks to be incorporated. For this dataset, a restricted covariance structure encapsulating such prior knowledge led to further performance improvements over a free-form covariance matrix, although the performance increase was smaller than the difference between multi- and single-task models. This provides clear evidence that the restricted task prior is appropriate for this dataset. However, a restricted prior is unlikely to be appropriate in all cases. In multi-output regression for example, it is common that the regression targets associated with different tasks are not calibrated across the same range. In such situations it is crucial to be able to model the task covariances quantitatively. Also, with a larger training dataset than was used here, it may be possible to estimate more subtle interactions between tasks. In such cases a more flexible model may also be preferred. In line with this interpretation, the contrasts that were more accurately predicted using the restricted covariance corresponded to those that were the most difficult to predict (Fig. 3), suggesting that the rigid covariance structure facilitated tasks borrowing strength from one another. More generally, an undirected graphical model could also be specified for the task covariance (see for example Sheldon (2008)), which could model more complex interactions such as session or site effects. As we have shown, this can be effectively combined with the automatic parameter optimisation provided by GP models to automatically fine-tune the structure of the task covariance matrix. A final benefit of using a flexible covariance matrix is that it may help to minimise the effect of “negative transfer”, which is known to be a challenge in MTL models. Negative transfer refers to the induction of coupling between tasks that are in fact unrelated, which may ultimately degrade performance rather than improving it (see Pan and Yang (2010) for further discussion). In a neuroimaging context, this may be useful to downweight subjects with a poor signal to noise ratio or an atypical response profile, that otherwise may degrade predictive accuracy for the other subjects.

Many neuroimaging studies focus on the predictive aspect of PR models, particularly for clinical applications. However, it is usually also important to interpret the coefficients of the model to quantify the contribution of each brain region to the predictions and to assess the reproducibility of model coefficients under perturbations to the data. This is most commonly done by mapping the classifier weight vector in the voxel space (e.g. Mourao-Miranda et al. (2005); Kloppel et al. (2008); Marquand et al. (2012); Gaonkar and Davatzikos (2012)). Since the weights alone do not determine the contribution of each voxel to the prediction, we propose to take this approach a step further by mapping the product of the weights and the data at each brain voxel. This “predictive mapping” approach is applicable to many of types of classifier employed in neuroimaging (e.g. support vector machines and penalised linear models) and yields two main benefits: (i) it is intuitively appealing in that it quantifies the total contribution of what was actually used to make predictions at each brain voxel and (ii) it lends itself naturally to a statistical approach to assess the predictive contribution of different brain regions. In the VAL v INVAL contrast we examined, the MTL models induced strong coupling between the tasks (i.e. subjects). As expected, this resulted in high within- and between subject reproducibility in the patterns of discriminative weights. In contrast, single-subject and pooled classification approaches yielded discriminative patterns that were either inconsistent across subjects or that showed poor reproducibility across cross-validation folds. This in turn led to an increase in both the magnitude of t-statistics and the number of voxels surviving an arbitrary but commonly used threshold in the predictive maps for the MTL- relative to STL models (Fig. 7). All PR approaches detected more significant voxels than a comparable univariate SPM, which provides an indication of the utility of predictive mapping for detecting spatially distributed effects. However, it is important to note that the SPMs and predictive maps have a different interpretation: the SPMs describe focal, group level effects; the predictive maps summarise the total contribution of each brain region to predicting the class labels at the single subject level. Correspondingly, the regions with a large group-level difference are not necessarily the same as those that are most useful for prediction. Another important caveat to the foregoing is that the degree of regularisation employed also influences the reproducibility of the spatial patterns (Rasmussen et al., 2011), so we cannot exclude the possibility that different degrees of regularisation between single- and multi- task learning models contributed to their differential effects. To investigate this, we repeated the STL analysis using a range of different regularisation strengths with the covariance function described in (Marquand et al., 2010), and obtained nearly identical results, which would seem to eliminate this possibility.

We argue that by accommodating inter-subject variations as different tasks and searching for commonalities between subjects, MTL models are better able to capture the consistent discriminative pattern across the group relative to the corresponding single-task models. This consensus pattern is usually what is of primary interest in multi-subject neuroimaging studies, and is probably responsible for the improvement in accuracy provided by MTL over STL models. This rationale also bears similarities with recent work that aims to select the most stable features for decoding cognitive states (Gramfort et al., 2011; Rondina et al., 2014; Ryali et al., 2012). In general, the magnitude of the improvement provided by coupling the subjects through MTL is likely to be dependent on the particular application and the nature of the pattern of responses elicited by the fMRI task. This is because the functional anatomy in some brain regions (e.g. frontal eye fields) is well aligned to cortical structures whereas the anatomical locations of other highly specialised regions (e.g. fusiform face area) are more variable across subjects, even after subjects have been well aligned structurally (Frost and Goebel, 2012). Similarly, the degree of spatial smoothing applied to the data may influence the similarity of the images belonging to different subjects. However, it is important to point out that the proposed method is still able to accommodate the settings where variability between subjects is high or the smoothing is not optimal by reducing the coupling induced between the tasks.

In this work, we framed the MTL problem in the context of GP models, which have desirable properties for neuroimaging (e.g. probabilistic predictions and the ability to automatically tune model hyperparameters using type-II maximum likelihood). Another important motivation for our choice of method was the scalability of the method to whole-brain voxel-wise data and large numbers of tasks. We expect that many of the benefits of MTL models we have demonstrated are not limited to GPs and may generalise to different MTL approaches. For further work, it would be interesting to evaluate approaches that confer different benefits, such as structured sparsity (e.g. Michel et al. (2011); Grosenick et al. (2013); Sohn and Kim (2012); Marquand et al. (2013a)). However, an important point to bear in mind is that for MTL it is necessary to estimate a large number of weight vectors (at least one per task). This may become problematic if the analytical method scales according to the dimensionality of the input space, as is often the case for sparse models. In contrast, the computational complexity of GPs and other kernel methods is governed by the number of samples and does not increase with increasing dimensionality. This implies that sparse models are probably suited to different types of problems (e.g. regional summary measures). On the other hand, a well-known limitation with GP models is an unfavourable cubic scaling in the number of data points. Therefore, for very large samples it may be necessary to use approximations to speed up computation. To provide an indication of the computational cost of the proposed methods on the dataset we examined, the most computationally expensive method proposed (MTL (F)) requires a few minutes to optimise hyperparameters and 1–2 s to make predictions for a dataset with 350 samples using a single 2.8 GHz CPU core.

Other avenues of future work include generalising the MTL models employed in this work to other likelihood functions, such as multi-class classification (Filippone et al., 2012) and ordinal regression (Doyle et al., 2013), integrating out the dependency on model hyperparameters using Markov chain Monte Carlo approaches and investigating the effect of preprocessing operations (e.g. spatial smoothing) on the accuracy of MTL models.

In summary, we have presented an empirical evaluation of MTL models for decoding multi-subject functional neuroimaging data. We have demonstrated that by providing a flexible framework to capture inter-subject variation in neuroimaging data, MTL confers two concrete benefits: (i) the potential to improve the accuracy of PR models in neuroimaging and (ii) more consistent representations of the discriminating patterns underlying the predictions relative to the single-task models most commonly used in current practice. Our results suggest that MTL is a promising method for neuroimaging data analysis.

The following are the supplementary data related to this article

Supplementary material Supplementary PDF document containing a didactic simulation study of multi-task learning and supplementary results.

Supplementary data to this article can be found online at http://dx.doi.org/10.1016/j.neuroimage.2014.02.008.

## Conflict of Interest

The authors have no conflict of interest to declare.

## Figures and Tables

**Table 1:** Experimental conditions for the evaluation dataset. See text for details. Abbreviations: HC = high confidence, LC = low confidence.

	Name	Description
1	Valid HC hit cue	Valid cue where following object was a subsequent high confidence hit
2	Valid LC hit cue	Valid cue where following object was a subsequent low confidence hit
3	Valid miss cue	Valid cue where following object was a subsequent miss
4	Invalid HC hit cue	Invalid cue where following object was a subsequent high confidence hit
5	Invalid LC hit cue	Invalid cue where following object was a subsequent low confidence hit
6	Invalid miss cue	Invalid cue where following object was a subsequent miss
7	Valid other cue	Valid cue where following stimulus was an untested or no response object
8	Valid other greeble cue	Valid cue where following stimulus was an untested or no response greeble
9	Invalid other greeble cue	Invalid cue where following stimulus was an untested or no response greeble
10	Valid HC-hit object	Object that was a subsequent high confidence hit and that followed a valid cue
11	Valid LC-hit object	Object that was a subsequent low confidence hit and that followed a valid cue
12	Valid miss object	Object that was a subsequent miss and that followed a valid cue
13	Invalid HC-hit object	Object that was a subsequent high confidence hit and that followed an invalid cue
14	Invalid LC-hit object	Object that was a subsequent low confidence hit and that followed an invalid cue
15	Invalid miss object	Object that was a subsequent miss and that followed an invalid cue
16	Valid other object	Stimulus that was an untested or no response object and that followed a valid cue
17	Valid greeble object	Stimulus that was an untested or no response greeble and that followed a valid cue
18	Invalid greeble object	Stimulus that was an untested or no response greeble and that followed a invalid cue

**Table 2:** Classification accuracy for each classifier on the illustrative contrasts under leave-one-run-out cross-validation. Models showing the best performance are highlighted in boldface and values in parenthesis are the standard error across 10 cross-validation folds. Abbreviations: MTL (F) = multi-task learning (free-form), MTL (R) = multi-task learning (restricted).

Contrast	Single subject	Pooled	MTL (F)	MTL (R)
CUE v OBJ	93.68 (1.44)	95.65 (1.08)	**97.00 (0.79)**	96.86 (0.80)
VAL v INVAL	66.70 (1.94)	69.09 (2.72)	70.64 (2.73)	**74.26 (1.55)**
HIT v MISS	67.57 (2.55)	67.11 (2.39)	**68.42 (1.94)**	**68.42 (1.74)**

**Table 3:** Area under the ROC curve for each classifier on the illustrative contrasts under leave-one-run-out cross validation. Models showing the best performance are highlighted in boldface. Abbreviations: MTL (F) = multi-task learning (free-form), MTL (R) = multi-task learning (restricted).

Contrast	Single subject	Pooled	MTL (F)	MTL (R)
CUE v OBJ	0.995	0.996	**0.999**	**0.999**
VAL v INVAL	0.657	0.755	0.793	**0.820**
HIT v MISS	0.706	0.702	**0.764**	0.750

**Table 4:** Summary of the proportion of classifiers for which MTL (R) afforded an advantage relative to the other baseline classification approaches across all 66 pairwise classifiers under leave-one-run-out cross-validation. P-values were determined by Wilcoxon signed rank test. Abbreviations: MTL (F) = multi-task learning (free-form), MTL (R) = multi-task learning (restricted).

Baseline method	MTL (R) > baseline (% contrasts)	Mean increase (% acc.)	MTL (R) < baseline (% contrasts)	Mean decrease (% acc.)	p-value
Single subject	77.27	5.39	22.73	− 2.91	6 × 10^− 7^
Pooled	74.24	4.18	21.21	− 1.46	3 × 10^− 7^
MTL (F)	59.09	2.13	37.88	− 0.01	0.004

**Table 5:** Classification accuracy for pooled and MTL (R) classifiers on the illustrative contrasts under leave-one-subject-out cross-validation. Models showing the best performance are highlighted in boldface and values in parenthesis are the standard error across 18 cross-validation folds. Abbreviations: MTL (R) = multi-task learning (restricted covariance).

Contrast	Pooled	MTL (R)
CUE v OBJ	88.90 (2.35)	**93.52 (1.73)**
VAL v INVAL	70.44 (2.25)	**72.14 (1.71)**
HIT v MISS	**66.30 (2.32)**	64.41 (1.31)

**Table 6:** Area under the ROC curve for pooled and MTL (R) classifiers on the illustrative contrasts under leave-one-subject-out cross validation. Models showing the best performance are highlighted in boldface. Abbreviation: MTL (R) = multi-task learning (restricted).

Contrast	Pooled	MTL (R)
CUE v OBJ	0.976	**0.996**
VAL v INVAL	0.759	**0.794**
HIT v MISS	0.709	**0.720**
