# Supplementary material for: Bayesian multi-task learning for decoding multi-subject neuroimaging data
Source: Neuroimage. 2014 May 15;92(100):298–311. doi: 10.1016/j.neuroimage.2014.02.008 (PMC4010954; doi:10.1016/j.neuroimage.2014.02.008)
Supplement: Supplementary material — Supplementary PDF document containing a didactic simulation study of multi-task learning and supplementary results. [file mmc1.pdf]

Supplementary Material for: Bayesian Multi-Task Learning for Decoding Multi-Subject Neuroimaging Data

Andre F. Marquand*[1], Michael Brammer[1], Steve C. R. Williams[1], and Orla Doyle[1]

[1]Department of Neuroimaging, Institute of Psychiatry, De Crespigny Park, London SE5 8AF, United Kingdom

January 28, 2014

1 Simulation study of multi-task learning

In this section, we present a small didactic simulation to illustrate the effect of task coupling on the predictive accuracy and weight vectors of Gaussian process multi-task learning (MTL) models. Note that we employ a highly simplified problem setting to clearly illustrate the mechanisms by which the transfer of information between tasks occurs under the proposed model. It is not intended to provide a realistic model for the expected noise properties of neuroimaging data. We employ a similar notation to that used in the main text, where the dataset consists of an $n_x \times d$ matrix of inputs, $\mathbf{X}$, and an n_y-vector of outputs, $\mathbf{y}$. We use $\mathbf{x}_i$ to denote individual input vectors and $\mathbf{x}_{ip}$ for the i-th sample in the p-th task if the input vectors for the tasks are distinct. We use $\mathbf{K}^f$ to denote the task covariance matrix, and $\mathbf{K}^x$ for the input covariance. For incomplete designs with n_y output values and m tasks, we define an $n_y \times m$ indicator matrix, $\mathbf{M}$, denoting task membership (see main text for details). This allows us to define $\mathbf{K}^F = \mathbf{M}\mathbf{K}^f\mathbf{M}^T$ for the incomplete task covariance. We also use $\mathbf{K}^X$ to describe the input covariance in an incomplete design, which is distinct from $\mathbf{K}^x$ in that it may have missing values or be based on duplicated inputs.

In the proposed Gaussian process MTL framework, tasks are coupled through both the inputs and the ouputs. We demonstrate both types of coupling in the simulation below. As described in Bonilla et al. (2008), another important property of the model is that there is a cancellation of inter-task transfer if: (i) the design is complete and (ii) the output noise is zero. Note that both criteria

*Corresponding author. andre.marquand@kcl.ac.uk

must be met for inter-task transfer to cancel. We also demonstrate this effect in the simulation below.

1.1 Simulation of a complete design

To illustrate the effects of task coupling under a complete design, we simulate a multi-output regression model with eight correlated outputs. We generate 20 input samples on a 20 dimensional input space (i.e. $\mathbf{x}_i \in \mathbb{R}^{20}, i = 1, \ldots, 20$). Each input vector is drawn from an isotropic zero-mean Gaussian distribution with standard deviation of 0.5. We embed two signals in these data: (i) a linear trend in the first two input dimensions, and (ii) a weak linear trend in the last two input dimension (corrupted with noise having a standard deviation of 0.2). We generate a task covariance matrix by sampling from an inverse Wishart distribution, $\mathbf{K}^f \sim \mathcal{IW}(\mathbf{\Psi}, \nu)$, with $\mathbf{\Psi} = 0.5\mathbf{1}_{8\times 8} + 0.5(8)\mathbf{I}_8$, and $\nu = 13$. By referring to equation (10) in the main text we see that that this generates a random covariance matrix having a moderate task coupling. Note that we use a degrees of freedom (ν) slightly higher than the number of tasks ($m = 8$) because the sampling variability of the inverse Wishart distribution is high with low degrees of freedom. In practice, this still allows the task covariance matrix to deviate substantially from the expected value of the distribution. We generate function space weight vectors for each task from a zero-mean isotropic Gaussian distribution with a standard deviation of 0.05 (i.e. $\boldsymbol{\alpha} = [\boldsymbol{\alpha}_1^T, ..., \boldsymbol{\alpha}_m^T]^T, \boldsymbol{\alpha}_p \sim \mathcal{N}(\mathbf{0}, 0.05\mathbf{I})$). We use a linear input covariance, $\mathbf{K}^x = \mathbf{X}\mathbf{X}^T$ and generate latent function values by $\mathbf{f} = (\mathbf{K}^f \otimes \mathbf{K}^x)\boldsymbol{\alpha}$. Finally, we add noise to the outputs by $\mathbf{y} = \mathbf{f} + \mathbf{n}$, where $\mathbf{n} \sim \mathcal{N}(\mathbf{0}, \sigma_n^2\mathbf{I})$. We split the data into a training and test portion (50% for each) and estimate noise parameters and a free-form task covariance from the training data as described in the main text. We make predictions on the test set using the standard GP predictive equations. We consider the case where $\sigma_n^2 = 0.1$, then consider the noise-free case ($\sigma_n^2 = 0$). In each case we report the results from 20 random repetitions of the above procedure and compare the results to a single task learning (STL) model, where each output is learned independently. We quantify predictive accuracy on the test set using the standardized mean squared error (SMSE; see Rasmussen and Williams (2006)) and also report representative examples of the predictive weights, computed using equation (22) in the main text.

For the case where $\sigma_n^2 = 0.1$, the MTL model obtained a mean (SEM) SMSE of 0.208 (0.038), and was slightly more accurate than the STL model, which obtained a mean SMSE of 0.245 (0.052). As expected, under the zero noise condition, the MTR and STR models obtained identical results, both achieving a mean SMSE = 0.124 (0.019).

The input space weight vectors are shown in Figure 1. Both the STL and MTL models learned high weights for the strong and weak linear trends. In a multi-output learning context, the effect of the task coupling on the weights is relatively subtle because the inputs for the different tasks are the same. For these data, the effect of inter-task coupling is largely restricted to constraining the magnitude of the weights to be similar for the different outputs. Two examples

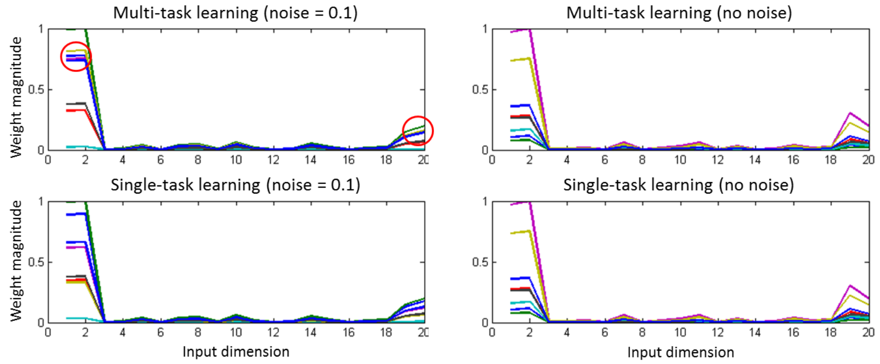

Figure 1: Predictive weights for a complete design/multi-output learning model, scaled such that the maximum weight across all tasks is equal to one. Left panels: models with noise variance equal to 0.1, right panels: noise free case. Each line represents the weight vector for a single task. Two examples of task coupling are indicated by circles.

of coupled weight vector coefficients are indicated by circles in Figure 1. In the noise-free case, the weight vectors are identical.

1.2 Simulation of an incomplete design

To illustrate the effects of task coupling under an incomplete design, we simulate a model where we predict eight correlated outputs from eight distinct sets of inputs. The simulation follows that described in the previous section; the main difference is that each of the outputs has a distinct set of inputs. For each task, p, we again construct 20 dimensional input vectors (i.e. $\mathbf{x}_{ip} \in \mathbb{R}^{20}, i = 1, \ldots, 20, p = 1, \ldots, 8, \mathbf{x}_{ip} \sim \mathcal{N}(\mathbf{0}, 0.5\mathbf{I})$. We embed signals within the inputs for each task such that the first and $p+1$-th dimensions contain a linear trend and the last two dimensions contain a weak linear trend, as above. Thus, the first and last two dimensions contain signal that is common to all tasks and there is another signal component that varies in location between tasks. We sample a task covariance matrix from the same inverse Wishart distribution as above and generate function space weights randomly, $\boldsymbol{\alpha}_p \sim \mathcal{N}(\mathbf{0}, 0.05\mathbf{I}))$. We generate latent function values by by $\mathbf{f} = (\mathbf{K}^F \odot \mathbf{K}^X)\boldsymbol{\alpha}$ and add noise to the outputs by $\mathbf{y} = \mathbf{f} + \mathbf{n}$, where $\mathbf{n} \sim \mathcal{N}(\mathbf{0}, \sigma_n^2\mathbf{I})$. As above, we consider both $\sigma_n^2 = 0.1$ and $\sigma_n^2 = 0$ under the same training and test split and report the results averaged over 20 random repetitions

For the case where $\sigma_n^2 = 0.1$, the MTL model obtained a mean (SEM) SMSE of 0.438 (0.140). This was more accurate than the STL model, which obtained a mean SMSE of 0.683 (0.187). In this case there is not a cancellation of task transfer in the noise-free case, because the design is incomplete. When $\sigma_n^2 = 0$, the MTL model achieved an SMSE of 0.013 (0.003). This was more accurate

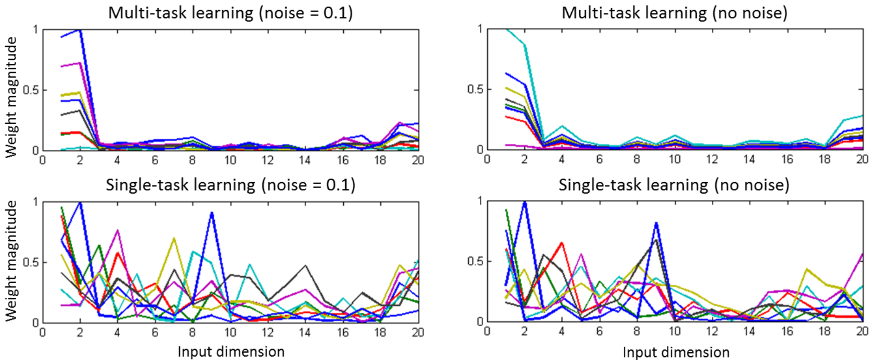

Figure 2: Predictive weights for an incomplete design, scaled such that the maximum weight across all tasks is equal to one. Left panels: models with noise variance equal to 0.1, right panels: noise free case. Each line represents the weight vector for a single task.

than the STL model, which achieved a mean SMSE of 0.088 (0.030).

The input space weight vectors for the incomplete design are shown in Figure 2. For illustrative purposes, we show an example of weights with strong coupling. The differences between the single- and multi-task approaches are more obvious in this case. At both noise levels considered, the MTL models concentrate weight primarily on the signal components that are shared between tasks. While the STL weight vectors also have high weight for these components, they show considerably more variation across tasks and also apply high weights to the other input dimensions. While some of these weights reflect the different signal properties of the individual tasks (e.g. dimension nine, which contains signal in the eighth task), they are also noisier (e.g. dimensions 10-18 are sometimes assigned non-zero weight despite containing no signal).

2 Supplementary Results

2.1 Raw accuracies for all pairwise contrasts

A numerical summary of the accuracy obtained by each of the classification approaches on all pair-wise contrasts is provided in Tables 1 - 6. Note that these results are also presented graphically in the main text.

2.2 Comparison of free-form multi-task learning with other approaches

A comparison of the MTL (F) classification approach with all other classification approaches is presented in Figure 3 and a high-level summary of these results

	1	2	3	4	5	6	7	8	9	10	11	12
1	-	-	-	-	-	-	-	-	-	-	-	-
2	53.19	-	-	-	-	-	-	-	-	-	-	-
3	56.93	55.72	-	-	-	-	-	-	-	-	-	-
4	58.77	57.11	62.12	-	-	-	-	-	-	-	-	-
5	59.23	52.02	61.24	52.95	-	-	-	-	-	-	-	-
6	57.01	54.73	60.40	56.93	57.58	-	-	-	-	-	-	-
7	94.21	93.74	93.51	91.60	92.17	95.78	-	-	-	-	-	-
8	88.14	88.40	88.57	85.82	85.30	88.52	59.90	-	-	-	-	-
9	95.00	94.99	94.44	90.83	87.79	96.94	64.75	53.76	-	-	-	-
10	86.67	86.60	86.02	77.81	83.27	86.89	69.67	65.76	71.15	-	-	-
11	82.28	83.10	83.46	82.49	75.11	82.97	65.99	59.79	65.78	58.67	-	-
12	90.80	90.20	89.14	86.20	87.68	91.53	70.88	63.75	66.57	59.06	58.30	-

Table 1: Accuracies for single subject classifiers on all pairwise contrasts (leave-one-run-out cross-validation). Row and column numbers index the different contrasts listed in Table 1 in the main text.

	1	2	3	4	5	6	7	8	9	10	11	12
1	-	-	-	-	-	-	-	-	-	-	-	-
2	50.36	-	-	-	-	-	-	-	-	-	-	-
3	57.46	51.11	-	-	-	-	-	-	-	-	-	-
4	54.85	52.78	57.72	-	-	-	-	-	-	-	-	-
5	50.54	51.15	54.54	50.64	-	-	-	-	-	-	-	-
6	52.62	55.47	60.49	49.89	48.43	-	-	-	-	-	-	-
7	95.56	95.87	97.66	90.50	90.35	94.26	-	-	-	-	-	-
8	93.34	91.82	95.80	87.11	84.90	93.51	61.45	-	-	-	-	-
9	95.83	94.72	96.94	89.09	84.17	94.37	66.19	51.85	-	-	-	-
10	93.46	90.55	93.34	87.04	82.92	91.22	69.81	60.75	69.14	-	-	-
11	89.11	91.66	90.70	86.30	83.79	87.53	68.64	61.55	66.61	52.56	-	-
12	95.56	94.10	96.67	86.48	86.75	93.82	74.10	63.50	70.00	57.60	55.97	-

Table 2: Accuracies for pooled classifiers on all pairwise contrasts (leave-one-run-out cross-validation). Row and column numbers index the different contrasts listed in Table 1 in the main text.

over all contrasts is presented in Table 7. Note that the contrast between MTL (F) and MTL (R) is presented in the main text. Similar to the MTL (R) method reported in the main text, the MTL (F) method performed significantly better overall than all single task learning approaches (Table 7).

	1	2	3	4	5	6	7	8	9	10	11	12
1	-	-	-	-	-	-	-	-	-	-	-	-
2	47.78	-	-	-	-	-	-	-	-	-	-	-
3	54.72	49.01	-	-	-	-	-	-	-	-	-	-
4	58.03	52.69	54.84	-	-	-	-	-	-	-	-	-
5	51.77	48.60	55.91	44.05	-	-	-	-	-	-	-	-
6	55.12	53.77	57.99	49.05	49.25	-	-	-	-	-	-	-
7	96.43	96.43	97.10	96.23	95.61	96.15	-	-	-	-	-	-
8	93.06	94.10	95.49	94.51	92.43	93.86	64.99	-	-	-	-	-
9	97.22	96.94	97.78	96.70	93.84	97.22	67.70	49.38	-	-	-	-
10	92.94	91.12	91.32	93.48	90.60	91.66	75.15	67.14	70.26	-	-	-
11	88.97	90.84	90.79	87.31	88.76	90.80	74.15	66.26	70.54	51.26	-	-
12	95.28	94.14	95.28	94.95	92.86	94.44	80.53	70.07	76.04	55.36	62.83	-

Table 3: Accuracies for MTL (F) classifiers on all pairwise contrasts (leave-one-run-out cross-validation). Row and column numbers index the different contrasts listed in Table 1 in the main text. Abbreviations: MTL (F) = multi-task learning with a free-form task covariance.

	1	2	3	4	5	6	7	8	9	10	11	12
1	-	-	-	-	-	-	-	-	-	-	-	-
2	51.91	-	-	-	-	-	-	-	-	-	-	-
3	57.46	49.72	-	-	-	-	-	-	-	-	-	-
4	60.66	56.91	61.04	-	-	-	-	-	-	-	-	-
5	55.12	48.14	57.04	49.08	-	-	-	-	-	-	-	-
6	56.63	56.48	59.24	53.27	50.05	-	-	-	-	-	-	-
7	97.10	96.79	97.66	97.13	96.31	96.83	-	-	-	-	-	-
8	92.91	93.86	94.14	93.51	93.44	94.41	64.44	-	-	-	-	-
9	97.50	97.22	97.78	97.26	94.53	96.94	71.03	51.85	-	-	-	-
10	92.39	90.47	90.67	92.22	90.34	90.98	75.41	66.77	74.31	-	-	-
11	89.11	89.76	89.09	86.69	86.90	88.32	79.27	68.83	72.60	54.74	-	-
12	94.17	94.14	94.44	94.91	91.83	93.89	82.87	73.84	76.88	59.96	57.84	-

Table 4: Accuracies for MTL (R) classifiers on all pairwise contrasts (leave-one-run-out cross-validation). Row and column numbers index the different contrasts listed in Table 1 in the main text. Abbreviations: MTL (R) = multi-task learning with a restricted task covariance.

2.3 Predictive voxel weights for each classification approach

Predictive weights for the single task learning approaches (single subject and pooled) are presented in Figures 4 and 5. These show that the single task learning approaches learned weights that were inconsistent across subjects, suggesting that they have focussed on the idiosyncratic properties of each subject.

	1	2	3	4	5	6	7	8	9	10	11	12
1	-	-	-	-	-	-	-	-	-	-	-	-
2	52.29	-	-	-	-	-	-	-	-	-	-	-
3	51.67	48.52	-	-	-	-	-	-	-	-	-	-
4	61.39	52.48	57.13	-	-	-	-	-	-	-	-	-
5	52.27	52.31	54.19	53.44	-	-	-	-	-	-	-	-
6	55.98	53.97	51.53	49.54	50.26	-	-	-	-	-	-	-
7	91.67	89.14	90.83	87.29	86.95	91.76	-	-	-	-	-	-
8	85.50	84.63	86.85	79.98	81.81	85.12	61.72	-	-	-	-	-
9	88.33	88.58	89.72	85.08	84.40	89.65	69.25	51.39	-	-	-	-
10	86.20	85.68	86.05	84.25	80.19	86.20	69.73	64.91	67.59	-	-	-
11	83.79	83.89	84.15	79.83	80.44	83.45	75.82	70.22	67.28	49.64	-	-
12	89.17	88.55	91.11	85.40	85.69	89.10	78.11	65.87	70.76	58.69	57.92	-

Table 5: Accuracies for pooled classifiers on all pairwise contrasts (leave-one-subject-out cross-validation). Row and column numbers index the different contrasts listed in Table 1 in the main text.

	1	2	3	4	5	6	7	8	9	10	11	12
1	-	-	-	-	-	-	-	-	-	-	-	-
2	50.65	-	-	-	-	-	-	-	-	-	-	-
3	50.99	48.77	-	-	-	-	-	-	-	-	-	-
4	62.76	53.12	57.92	-	-	-	-	-	-	-	-	-
5	53.84	54.71	54.64	52.17	-	-	-	-	-	-	-	-
6	60.45	51.17	55.00	51.44	50.34	-	-	-	-	-	-	-
7	94.33	94.05	94.33	94.09	93.02	94.33	-	-	-	-	-	-
8	90.96	91.36	91.36	91.47	90.06	93.30	65.31	-	-	-	-	-
9	94.44	95.56	94.44	94.07	91.36	94.72	69.13	51.39	-	-	-	-
10	88.54	88.64	87.26	89.67	87.71	89.29	70.90	65.47	68.63	-	-	-
11	85.31	85.99	84.97	84.55	83.34	85.06	76.44	69.84	69.56	62.50	-	-
12	91.67	91.88	91.67	90.95	90.48	92.50	80.46	69.38	76.04	61.30	63.30	-

Table 6: Accuracies for MTL (R) classifiers on all pairwise contrasts (leave-one-subject-out cross-validation). Row and column numbers index the different contrasts listed in Table 1 in the main text. Abbreviations: MTL (R) = multi-task learning with a restricted task covariance.

The pooled models also showed poor reproducibility between cross-validation folds (see main text). In contrast, the coupling between the tasks induced by the multi-task learning framework caused the MTL classifiers to learn a consistent discriminative pattern across all subjects (Figures 6 and 7). Note that the coupling between tasks for the MTL (R) classifier was extremely strong and the weight vectors for the different tasks were virtually identical. As a result, only an example image is shown (Figure 7).

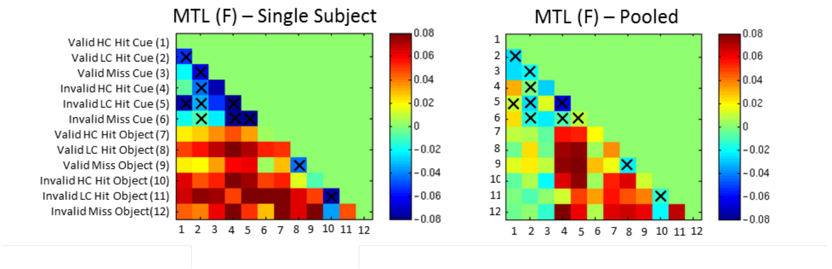

Figure 3: Difference in classification accuracy of MTL (F) in relation to single task learning classifiers for all pair-wise contrasts. The comparison between MTL (F) and MTL (R) is presented in the main text. Crosses denote comparisons for which the the classifier did not exceed 60% accuracy. Abbreviations: HC = high confidence, LC = low confidence, MTL = multi-task learning

Baseline method	MTL (F) > baseline (% contrasts)	mean increase (% acc.)	MTL (F) < baseline (% contrasts)	mean decrease (% acc.)	p-value
Single subject	71.21	5.59	28.79	-4.72	7×10^{-4}
Pooled	66.67	3.71	33.33	-1.68	3×10^{-4}

Table 7: Summary of the proportion of classifiers for which MTL (F) afforded an advantage relative to the other baseline classification approaches across all 66 pairwise classifiers (leave-one-run-out cross-validation). Abbreviations: MTL = multi-task learning

References

Bonilla, E. V., Chai, K. M., Williams, C. K. I., 2008. Multi-task Gaussian process prediction. In: Platt, J. C., Koller, D., Singer, Y., Roweis, S. (Eds.), Advances in Neural Information Processing Systems 20. MIT Press, Cambridge, MA, pp. 153–160.

Rasmussen, C., Williams, C. K. I., 2006. Gaussian Processes for Machine Learning. The MIT Press, Cambridge, Massachusetts.

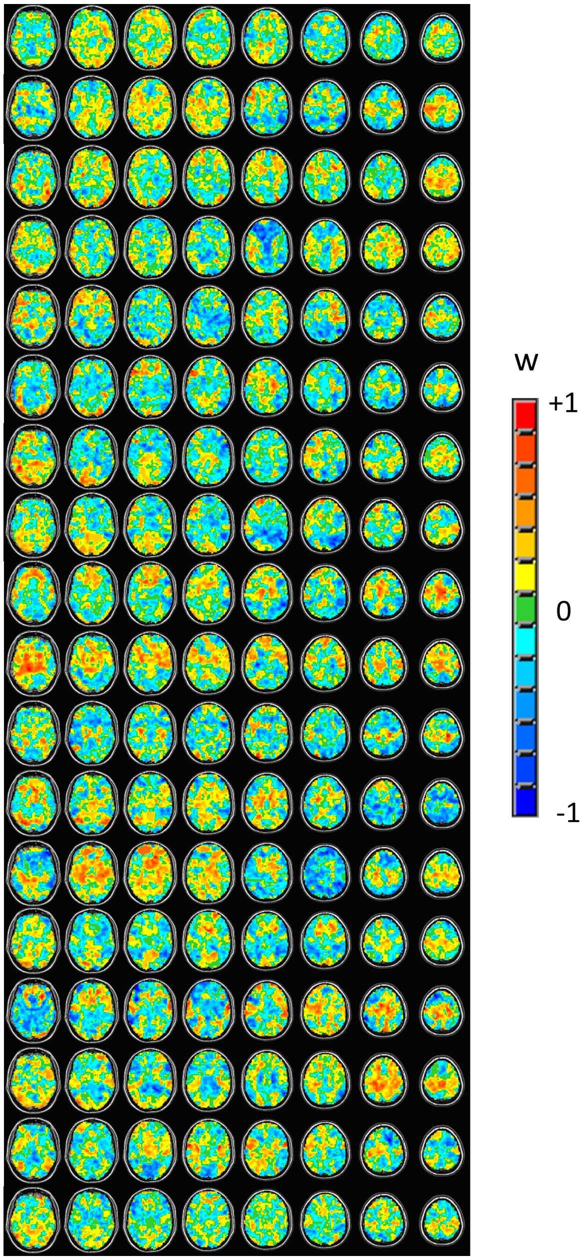

Figure 4: Predictive weights for the single subject classifiers (leave-one-run-out cross-validation). Each row represents a different subject. To assist visualisation, each row has been scaled such that the maximum weight magnitude is equal to one.

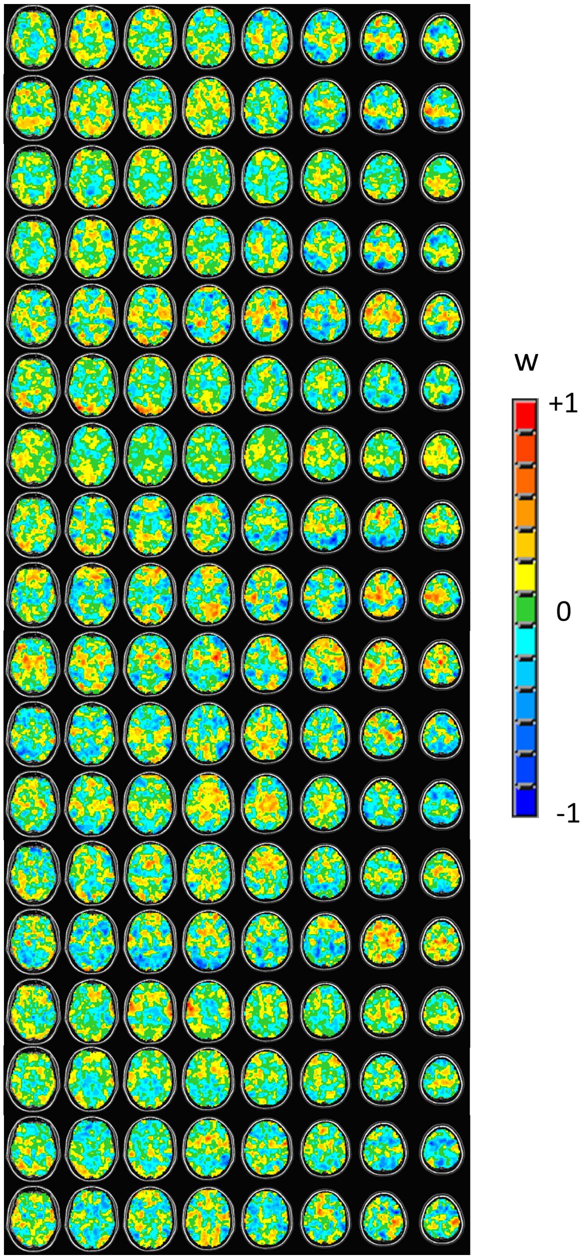

Figure 5: Predictive weights for the pooled classifiers (leave-one-run-out cross-validation). Each row represents a different subject. To assist visualisation, each row has been scaled such that the maximum weight magnitude is equal to one.

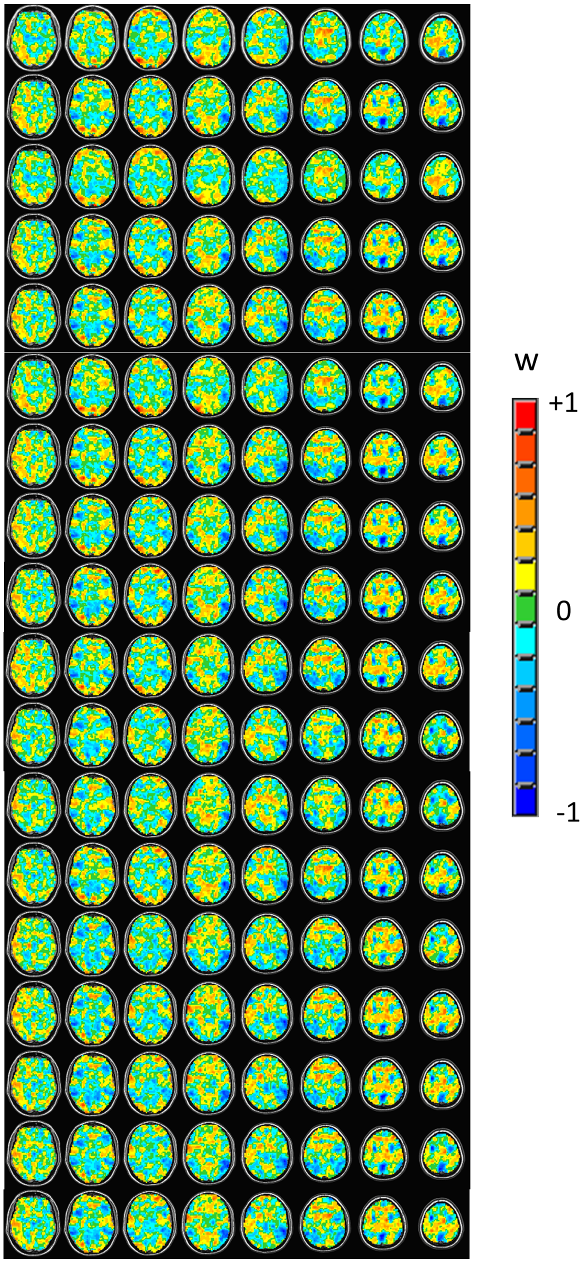

Figure 6: Predictive weights for the MTL (F) classifier (leave-one-run-out cross-validation). Each row represents a different subject (i.e. task). To assist visualisation, each row has been scaled such that the maximum weight magnitude is equal to one.

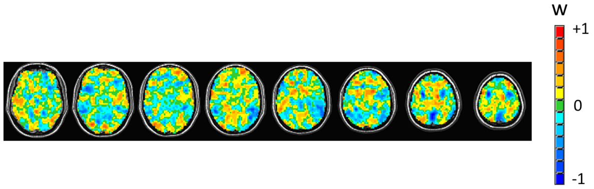

Figure 7: Example of predictive weights for the MTL (R) classifier (leave-one-run-out cross-validation). To assist visualisation, the image has been scaled such that the maximum weight magnitude is equal to one.
